# Reverse Engineering the Intracellular Self-Assembly of a Functional Mechanopharmaceutical Device

**DOI:** 10.1038/s41598-018-21271-7

**Published:** 2018-02-13

**Authors:** Tehetina Woldemichael, Rahul K. Keswani, Phillip M. Rzeczycki, Mikhail D. Murashov, Vernon LaLone, Brian Gregorka, Joel A. Swanson, Kathleen A. Stringer, Gus R. Rosania

**Affiliations:** 1 Biophysics Program, College of Literature, Science, and the Arts, University of Michigan, Ann Arbor, MI USA; 2 Department of Pharmaceutical Sciences, College of Pharmacy, University of Michigan, Ann Arbor, MI USA; 3 CLCI: Center for Live-Cell Imaging, Department of Microbiology and Immunology, University of Michigan, Ann Arbor, MI USA; 4 Program in Immunology and Department of Microbiology and Immunology, University of Michigan Medical School, Ann Arbor, MI USA; 5 Department of Clinical Pharmacy, College of Pharmacy, University of Michigan, Ann Arbor, MI USA

## Abstract

Weakly basic, poorly soluble chemical agents could be exploited as building blocks for constructing sophisticated molecular devices inside the cells of living organisms. Here, using experimental and computational approaches, we probed the relationship between the biological mechanisms mediating lysosomal ion homeostasis and the self-assembly of a weakly basic small molecule building block (clofazimine) into a functional, mechanopharmaceutical device (intracellular Crystal-Like Drug Inclusions – “CLDIs”) in macrophage lysosomes. Physicochemical considerations indicate that the intralysosomal stabilization of the self-assembled mechanopharmaceutical device depends on the pH_max_ of the weakly basic building block and its affinity for chloride, both of which are consistent with the pH and chloride content of a physiological lysosomal microenvironment. Most importantly, *in vitro* and *in silico* studies revealed that high expression levels of the vacuolar ATPase (V-ATPase), irrespective of the expression levels of chloride channels, are necessary and sufficient to explain the cell-type dependent formation, stabilization, and biocompatibility of the self-assembled mechanopharmaceutical device within macrophages.

## Introduction

Clofazimine (CFZ) is a weakly basic, poorly soluble, FDA-approved small molecule antibiotic that self-assembles^1,2^ into insoluble, biocrystalline structures known as Crystal Like Drug Inclusions (CLDIs) in macrophages of both mice and humans^3–5^. In mice, CFZ precipitates and accumulates in tissue macrophages as a biocrystalline hydrochloride salt (CFZ-H^+^Cl^−^). Remarkably, in Kupffer cells, splenocytes, alveolar macrophages, and peritoneal macrophages, the amount of intracellular CFZ-H^+^Cl^−^ typically exceeds that of every other cellular component except water^5^. Known factors that contribute to this massive bioaccumulation and self-assembly phenomenon include the drug’s high oral bioavailability, coupled with large daily doses, prolonged treatment regimens, and long elimination half-life^6^.

The observed self-assembly, mechanical, and optical properties^7^ of CFZ-H^+^Cl^−^, which can function as drug depots and photoacoustic contrast agents in macrophages, inspired our thinking about reverse engineering this weakly basic, small molecule chemical agent as a molecular building block for constructing other kinds of functional mechanopharmaceutical devices. Such devices could be used to influence the mechanical properties of cells. They could also be exploited as bio-responsive pharmaceutical or imaging agents by capitalizing on both physical and chemical interactions between cells and the self-assembled biocrystalline structures, *in vivo*^3,5,8^. In macrophages, CFZ-H^+^Cl^−^ forms elastic structures, which bend and relax in response to biomechanical forces^7^. Furthermore, the presence of these mechanopharmaceutical devices inside macrophages is associated with augmented anti-inflammatory activity^9,10^: upon phagocytosis, they lead to changes in cellular function, which include alterations in signal transduction pathways that affect the cell’s inflammatory response^10^. Unlike the soluble form of CFZ, which tends to be cytotoxic, the phagocytosis of the self-assembled mechanopharmaceutical device does not lead to toxicity^8,11^.

Thus, given that macrophages express high levels of proton pumping vacuolar ATPase (V-ATPase) and chloride channels on their lysosomal membranes^12–14^, and that weak bases are prone to accumulate inside lysosomes^15^, we probed whether the expression levels of these lysosomal membrane proteins in macrophages actively drive the accumulation of CFZ-H^+^Cl^−^ and its self-assembly in these cells. Using a well-established and published lysosomal ion regulation model^16^, we studied how proton pumping and chloride transport mechanisms influenced cell-type specific stabilization of CFZ-H^+^Cl^−^ inside lysosomes. In addition to verifying model predictions and further refining our understanding of the computational simulation results, we performed experiments using pharmacological inhibitors of V-ATPase^17^ and chloride channels^18^ to probe the biological mechanism underpinning the massive accumulation and self-assembly of the building block.

## Materials and Methods

### Solubility measurements

Freeze-dried CFZ-H^+^Cl^−^ ^11^ samples were weighed and 25 mg was added to each of five scintillation vials. Mili-Q water (15 mL) was added to each vial to ensure that CFZ-H^+^Cl^−^ crystals were in great excess. An aliquot of 0.1 M NaOH was added to each vial to achieve the initial equilibration pH measurements as follows: sample vials 1–5 initially contained 0, 40, 80, 120, and 200 µL of 0.1 M NaOH solution, respectively. After a 24-hour equilibration period, 10 µL of 0.1 M NaOH was added each day for a period of five days resulting in pH range of 4.5 to 8.9. The sample vials were placed on a magnetic stirrer plate in a 25 °C water bath. Each sample was allowed to equilibrate for at least 24 hours, after which 500 µL of sample was removed and filtered through a Spin-X centrifuge tube filter (0.45 μm cellulose acetate, 2 mL polypropylene tubes, non-sterile, Costar®, Cat # 8163) for 4 min @ 10,000 rpm. The pH of the filtered sample was determined (UltraBasic pH meter, Denver Instrument, Bohemia, NY), after which the sample was subjected to HPLC analysis (see Supplementary Methods) (Waters Alliance, Separations Module 2695) to determine the total solubility of the drug at the measured pH. For each sample, solubility measurement was performed in triplicate, and the average was used to construct the total drug solubility-pH profile. The standard curve was generated using CFZ-H^+^Cl^−^ crystals that were dissolved in the mobile phase at known concentrations (1–50 µM).

### Thermodynamic equilibrium model of CFZ’s solubility

For weak electrolytes, such as weakly basic drugs, the protonation state of the drug (B) is dictated by the relationship between the drug’s association constant (*K*_a_) and the acidity of its environment (H_3_O^+^). This is represented by the following equilibrium expression:

1 $${\rm{B}}+{{\rm{H}}}_{3}{{\rm{O}}}^{+}\rightleftarrows {{\rm{BH}}}^{+}+{{\rm{H}}}_{2}{\rm{O}}$$

where BH^+^ is the protonated form of the drug as a result of the interaction of the free base form of the drug with hydronium ion, and H_2_O is the byproduct of the reaction and remains constant. Thus, the equilibrium constant (*K*) is multiplied by H_2_O to obtain *K*_a_. Therefore, equation (1) is re-written using *K*_a_.

2 $${\rm{B}}+{{\rm{H}}}_{3}{{\rm{O}}}^{+}\rightleftarrows {{\rm{BH}}}^{+}$$

The mass law equilibrium equation is written as the following, assuming an ideal solution where the activity of a given species equals the concentration of the species (represented by the brackets):

3 $${K}_{a}=\frac{[{{\rm{BH}}}^{+}]}{[{\rm{B}}][{{\rm{H}}}_{3}{{\rm{O}}}^{+}]}$$

By writing the logarithmic form of equation (3), we have the following relationship, which is the Henderson-Hasselbach equation:

4 $${\rm{pH}}-{\rm{p}}{K}_{a}=\,\mathrm{log}\,\frac{[{\rm{B}}]}{[{{\rm{BH}}}^{+}]}$$

The equation is re-written as the following to express the concentrations of both the neutral and ionized forms of the drug:

5 $$[{\rm{B}}]=[{{\rm{BH}}}^{+}]\times {10}^{{\rm{pH}}-{\rm{p}}{K}_{a}}$$

6 $$[{{\rm{BH}}}^{+}]=[{\rm{B}}]\times {10}^{{\rm{p}}{K}_{a}-{\rm{pH}}}\,$$

According to thermodynamic laws of mass action, the total amount of drug is comprised of both the neutral and ionized forms at any given pH of its environment^19^. Thus, the total solubility (S_T_) of the drug is written as:

7 $${{\rm{S}}}_{{\rm{T}}}=[{\rm{B}}]+[{{\rm{BH}}}^{+}]$$

However, depending on the pH of the solution, the total drug solubility equation must be slightly modified to account for the distinction of the primary species in the solid versus solution phase^19^. For pH < pH_max_, the ionized form of the drug is the saturated species that is in equilibrium with the salt from of the drug in the solid phase; therefore, its solubility remains constant while the solubility of the neutral form of the drug varies with respect to pH. Therefore, by substituting equation (5) into equation (7), the total drug solubility for pH < pH_max_ is re-written as:

8 $${{\rm{S}}}_{{\rm{T}}}={[{{\rm{BH}}}^{+}]}_{{\rm{S}}}\times (1+{10}^{{\rm{pH}}-{\rm{p}}{K}_{a}})$$

where the solid phase is denoted by the subscript “s”.

To the contrary, for pH > pH_max_, the neutral form of the drug is in the solid phase; thus, its solubility remains constant whereas the solubility of the ionized form of the drug varies with respect to pH. Therefore, by substituting equation (6) into equation (7), the total solubility for pH < pH_max_ can be re-written as:

9 $${{\rm{S}}}_{{\rm{T}}}={[{\rm{B}}]}_{{\rm{S}}}\times (\,1+{10}^{{\rm{p}}{K}_{a}-{\rm{pH}}})$$

Moreover, at pH = pH_max_, both the ionized and neutral forms of the drug are the saturating species, where both the salt and free base precipitate forms are in the solid phase. Thus, equation (7) can be re-written as:

10 $${{\rm{S}}}_{{\rm{T}}}={[{\rm{B}}]}_{{\rm{S}}}+{[{{\rm{BH}}}^{+}]}_{{\rm{S}}}$$

For our purpose, we substituted our model drug CFZ in the above equations (1–10). Rewriting equation (7) results in the following total CFZ-H^+^Cl^−^ solubility equation:

11 $${{\rm{S}}}_{{\rm{T}}}={[{\rm{CFZ}}]}_{{\rm{S}}}+[{{\rm{CFZH}}}^{+}]$$

where the solid phase, which in this case is the free form of CFZ, is denoted by the subscript “s”, and the ionized form of the drug, [CFZH^+^], is in the solution phase, and similar to equation (6), is dictated by p*K*_a_ and pH as follows:

12 $$[{{\rm{CFZH}}}^{+}]={[{\rm{CFZ}}]}_{{\rm{S}}}\times {10}^{{\rm{p}}{K}_{a}-{\rm{pH}}}$$

Thus, by substituting equation (12) into equation (11), we obtain the following:

13 $${{\rm{S}}}_{{\rm{T}}}={[{\rm{CFZ}}]}_{{\rm{S}}}\times (1+{10}^{{\rm{p}}{K}_{a}-{\rm{pH}}})$$

Furthermore, by using different combinations of any two solubility-pH data points from the experimental total solubility-pH measurements, the p*K*_a_ and the intrinsic free base solubility values were simultaneously solved using equation (13). Then, by substituting these values into equation (13), the total CFZ-H^+^Cl^−^ solubility was calculated for the given range of pH 4.5 to 8.9, and the experimentally obtained total drug solubility-pH curve was fitted. Furthermore, in order to generate the total solubility-pH dataset for pH below pH_max_, we used the following equation:

14 $${{\rm{S}}}_{{\rm{T}}}=[{\rm{CFZ}}]+{[{{\rm{CFZH}}}^{+}]}_{{\rm{S}}}$$

Similar to equation (8), equation (14) can be further expressed as:

15 $${{\rm{S}}}_{{\rm{T}}}={[{{\rm{CFZH}}}^{+}]}_{{\rm{S}}}\times (1+{10}^{{\rm{pH}}-{\rm{p}}{K}_{a}})$$

However, we first had to determine [CFZH^+^]_S_. Because we only used one form of the drug (CFZ-H^+^Cl^−^ salt) as the starting material in the experimental measurements of total drug solubility as a function of pH, we used a mathematical proof approach to determine [CFZH^+^]_S_ and thereby pH_max_ (see Supplementary Methods) without necessarily having to experimentally generate a total drug solubility-pH curve for pH below pH_max_.

Moreover, the salt solubility product (*K*_sp_) of CFZ-H^+^Cl^−^ is given by the following equation:

16 $${K}_{sp}={[{{\rm{CFZH}}}^{+}]}_{{\rm{S}}}\times [{{\rm{Cl}}}^{-}]$$

However, because of the 1:1 stoichiometric relationship of CFZH^+^ and Cl^−^ in CFZ-H^+^Cl^−^ ^7^, the *K*_sp_ of CFZ-H^+^Cl^−^ in an aqueous media can be expressed as following:

17 $${K}_{sp}={({[{{\rm{CFZH}}}^{+}]}_{{\rm{S}}})}^{2}$$

### Animal experiments

Mice (4 week old, male C57BL/6J) were purchased from the Jackson Laboratory (Bar Harbor, ME) and acclimatized for 1 week in a specific-pathogen-free animal facility. Clofazimine (CFZ) (C8895; Sigma, St. Louis, MO) was dissolved in sesame oil (Shirakiku, Japan) to achieve a concentration of 3 mg/mL, which was mixed with Powdered Lab Diet 5001 (PMI International, Inc., St. Louis, MO) to produce a 0.03% drug to powdered feed mix, which was orally administered *ad libitum* for up to eight weeks. A corresponding amount of sesame oil was mixed with chow for vehicle treatment (control). Mice were euthanized via CO_2_ asphyxiation and exsanguination. Animal care was provided by the University of Michigan’s Unit for Laboratory Animal Medicine (ULAM), and the experimental protocol was approved by the Committee on Use and Care of Animals (Protocol PRO00005542). All animal experiments were done according to the protocol guidelines.

### Alveolar macrophage isolation

Following euthanasia, the trachea was surgically exposed and cannulated with a 20 G needle, and the lungs were lavaged by instilling 1 mL DPBS (Life Technologies) containing 0.5 mM EDTA (Sigma) six times. Approximately 90% of the instilled bronchoalveolar lavage (BAL) was retrieved. BAL was centrifuged (10 min at 400 × g, 4 °C), the supernatant removed, and the cell pellet was resuspended in RPMI 1640 media (Life Technologies) with 5% FBS (Life Technologies) and Penicillin/Streptomycin (Thermofisher). Cells were plated onto 4 or 8 chamber coverglass (#1.5, Lab-Tek II, Nunc, Rochester, NY) in RPMI for imaging studies. The cells were allowed to attach overnight, washed, and imaged in fresh RPMI.

### Peritoneal macrophage isolation

Following euthanasia, a small incision was made in the lower abdomen. The peritoneal cavity was then flushed with 10 mL of ice cold DPBS containing 5% FBS (Sigma) and collected. The peritoneal lavage was centrifuged for 10 min at 400 × g, 4 °C, and then re-suspended in DMEM media (Life Technologies) with 5% FBS and Penicillin/Streptomycin and counted. The cells were plated into Mat-tek dishes overnight in a serum-free growth media, and washed five times with phosphate-buffered saline (PBS).

### Macrophage depletion

Mice were fed CFZ or control diet continuously for a four-week period. Following two weeks of feeding, mice were treated with either liposomes containing either 7 mg/mL of clodronate or PBS (FormuMax Scientific Inc., Sunnyvale, CA) for two weeks. Mice were initially treated with 200 µL of liposomes followed by 100 µL injections twice per week (or a matching volume of PBS) to ensure continual macrophage depletion. Liposomes were injected intraperitoneally, as previously described^20^. After completing four weeks of feeding and two weeks of liposome treatment, mice were sacrificed and tissues were collected.

### Biochemical analysis of CFZ in tissues

The concentration of CFZ in the organs of mice was determined spectrophotometrically. After four weeks of CFZ- or vehicle-diet treatment, mice were euthanized via CO_2_ asphyxiation, and organs were collected. Tissue (20–30 mg) was homogenized in 500 µL of radioimmunoprecipitation assay buffer (Sigma) with added protease inhibitors (Halt protease and phosphatase inhibitor cocktail and 0.5 M EDTA; Thermo Pierce, Rockford, IL). Drug was extracted from homogenate (350 µL) with three washes with xylenes (1 mL). The drug was then extracted from the xylene with three 1 mL passes of 9 M sulfuric acid. The concentration of CFZ present in the tissue was determined using a 96-well plate reader (Biotek Synergy 2, Winooski, VT) (wavelength 540 nm). To account for extraction yield, untreated liver and spleen samples were spiked with known amounts of CFZ prior to extraction, and were analyzed in the same plate as the CFZ treated samples. The mass of CFZ per organ was determined using the yield-corrected concentration of CFZ in the mass of tissue analyzed, as determined through use of a standard curve with known concentrations of CFZ, and is reported as mg CFZ/g tissue.

### Biochemical analysis of CFZ in plasma

Blood was collected and centrifuged (7,000 × g for 5 minutes). The resulting supernatant serum was extracted with acetonitrile (90% extraction efficiency) for 10 min at 4 °C with vortexing. After centrifugation (15,000 rpm, 4 °C), the supernatant was injected into a Waters Acquity UPLC H-Class (Waters, Milford, MA) equipped with an Acquity UPLC BEH C18 column (1.7 μm, 2.1 mm [inner diameter] by 100 mm; Waters, Milford, MA). Mobile phase A was 5 mM ammonium acetate, adjusted to pH 9.9 with ammonium hydroxide, and mobile phase B was acetonitrile. The flow rate was 0.35 ml/min, with a linear gradient from 50 to 100% phase B over 1.5 min, followed by holding at 100% for 1.5 min, a return to 50% phase B, and then re-equilibration for 2.5 min. Standards were prepared by spiking untreated plasma samples with known amounts of clofazimine, ranging from 0 to 30 µM. Peak area was determined using Empower 3 Software (Waters, Milford, MA).

### Sample preparation for microscopy

In preparation for cryosectioning, portions of each organ were removed, immediately submerged in OCT (Tissue-Tek catalog no. 4583; Sakura), and frozen (−80 °C). Cryosectioning (5 µm) was carried out using a Leica 3050 S Cryostat (Leica Biosystems Inc., Buffalo Grove, IL). Immunohistochemistry of F4/80 (Abcam, 1:500 dilution) was performed using Alexa-Fluor 488 (Abcam, 1:500 dilution).

### Deep-etch, freeze-fracture electron microscopy

At the time of euthanasia, the liver was collected and kept cold (4 °C). The organ was frozen against a copper block, cooled with liquid helium, and stored in liquid nitrogen. The sample was fractured with Balzers 400 nitrogen cooled vacuum evaporator and freeze-etched for two minutes (−100 °C). A rotary replica was generated with 2 nm platinum and backed with 10 nm carbon film support. It was cleaned with chromo-sulfuric cleaning solution (Fisher Scientific, cat# SC88) for twelve hours and rinsed with DI water. The sample was picked up on formvar coated grids and viewed on a JEOL 1400 electron microscope with an AMT camera (JEOL USA, Inc., Peabody, MA).

### Spectral confocal microscopy

For the preparation of slides, CFZ-H^+^Cl^−^ crystals suspended in PBS (20 µl) were placed on a glass slide and a cover-slip was applied onto the sample prior to imaging. Spectral confocal microscopy was performed on a Leica Inverted SP5X confocal microscope system with two-photon FLIM (Leica Microsystems, Buffalo Grove, IL) using excitation wavelengths (470–670 nm). Image analysis and quantification were performed on Leica LAS AF. Several regions of interest of individual crystals were used to obtain fluorescence data, which were imported into MS-Excel for further analysis. All fluorescence yields were normalized to the maximum fluorescence yield measured across the tested spectral range, and background subtracted using data obtained from a blank slide.

### Epifluorescence microscopy of cells incubated with CFZ and Lysotracker® Blue

Macrophage-derived RAW264.7 cells (ATCC, Manassas, VA, ATCC Number: TIB-71^TM^) at a high seeding density of 100,000 cells/well, were grown in 8-chamber multiwell plates (Lab-Tek® II, Nunc, Rochester, NY) in Dulbecco’s Modified Eagles Medium (DMEM) + 10% Fetal Bovine Serum (FBS) + 1% Penicillin/Streptomycin (P/S) (500 µl/well growth media); cells were pre-incubated with CFZ (20 µM) in DMSO for 24–72 hours. For lysosomal confirmation, RAW264.7 cells were seeded at 30,000 cells/well in an 8-chamber multiwell plate (500 µl/well growth media). Twenty-four hours later, the growth media was replaced with media containing varying concentrations (0, 1, 10, 20 µM) of Lysotracker® Blue DND-22 (Thermo Fisher Scientific, Waltham, MA, Catalog No. L7525, excitation/emission maxima ~373/422 nm) and CFZ (10 µM). Visualization of all samples (cells or crystals) was done on a Nikon Eclipse T*i* (Nikon Instruments, Melville, NY). Fluorescence filters (excitation/emission) were optimized for 4,6-diamidino-2-phenylindole dihydrochloride (DAPI) (350/405 nm, exposure - 550 ms, *violet*), fluorescein isothiocyanate (FITC) (490/510 nm, exposure - 100–500 ms, *green*), Texas Red (590/610 nm, exposure - <500 ms, *red*), and Cy5 (640/670 nm, exposure - 500 ms, *far-red*). The Pearson’s Co-localization Coefficient (PCC) was computed for each individual cell as ROIs using the scatterplot distribution within the Nikon Elements AR software. Brightfield color photographs were acquired using a Nikon DS-Fi2 camera, whereas fluorescence photographs were acquired using a Photometrics CoolSNAP^TM^ *MYO* (Photometrics, Tucson, AZ) camera.

### Quantitative cytometric analysis

Following macrophage staining, the population of macrophages was determined by taking the ratio of the total F4/80 signal to the total nuclear signal across an image. Because of the unique Cy5-specific fluorescence property of CLDIs^11^, the prevalence of CLDIs was determined by taking the ratio of the total Cy5 signal to the total nuclear signal across an image. 5 images per mouse per organ were analyzed.

### Drug stability in a lysosomal microenvironment

CLDIs were isolated from spleen and liver of 8-week CFZ treated mice based on previously published protocols^10,21^. Free base CFZ crystals were pure CFZ crystals (Sigma-Aldrich, C8895). A speck (≪1 mg) of isolated CLDIs, CFZ-H^+^Cl^−^ crystals, and free base CFZ crystals were placed in 2 mL of each of the stability testing medias: 1× PBS (pH 7.4), lysosomal buffer without sodium chloride (LB-, pH 4.5), and lysosomal buffer with sodium chloride (LB+, pH 4.5). Lysosomal buffers were prepared according to a previously published protocol^22^. Samples were stirred in media for 5–7 days; after which the stability of crystals from each sample was monitored using brightfield and fluorescence microscopy using a Nikon Eclipse Ti inverted microscope (Nikon Instruments, Melville, NY). Look-up-tables (LUTs) and exposure times were maintained the same throughout the samples.

### Multi-parameter microscopy

Multi-parameter polarization, brightfield, and fluorescence microscopy was conducted using a Nikon Eclipse Ti inverted microscope (Nikon Instruments, Melville, NY). Polarization microscopy was performed using the LC-PolScope^23^, with the illuminating light narrowed to 623 nm by an interference filter (623 ± 23 nm, Semrock Optics, Rochester, NY). Polarization images were captured using an Abrio imaging system (Cambridge Research & Instrumentation, Inc, Woburn, MA). Brightfield images were captured using the Nikon DS-3 camera (Nikon Instruments) and fluorescence images were taken with the Photometrics CoolSnap MYO camera system (Photometrics, Tuscon, AZ) under the control of Nikon NIS-Elements AR software (Nikon Instruments). Illumination for fluorescence imaging was provided by the X-Cite 120Q Widefield Fluorescence Microscope Excitation Light Source (Excelitas Technology, Waltham, MA). Images were acquired and analyzed as previously described^24^.

### Raman sample preparation and measurements

Isolated mouse alveolar macrophages were transferred onto pre-sterilized silicon chips (16008; Ted Pella, Inc., Redding, CA) and incubated for 1 h (@ 37 °C and 5% CO_2_) to allow adherence to chip. The cell-containing chips were washed by brief submersion into isotonic NaCl (0.9%) solution followed by DI water, then allowed to air-dry. Raman measurements were acquired with the WiTec alpha300R confocal Raman microscope (WITec, Ulm, Germany) equipped with two excitation lasers: a 532 nm solid-state sapphire and a 785 nm wavelength-stabilized diode (0–55 mW and 0–88 mW tunable intensity ranges, respectively). A 100X air objective (Zeiss Epiplan-NEOFLUAR, NA (numerical aperture) = 0.9) coupled to a CCD detector via a multi-mode fiber of 100 μm diameter serving as the confocal pinhole, produced 0.72 μm and 1.06 μm illumination spots (for 532 nm and 785 nm lasers respectively). To minimize fluorescence background from pure CFZ and CFZ-H^+^Cl^−^ reference crystals, samples were excited with 785 nm. The 532 nm laser was utilized for excitation of biological samples due to its elicitation of a stronger Raman signal from the microscopic inclusions/CLDIs of interest. Point spectra were acquired (n ≥ 40 cells/group) by focusing a laser spot on cytoplasmic inclusions, CLDIs, or pure reference crystals: at each point, the laser was tuned to optimum intensity, and acquired point spectra over an integration time of 25 seconds. Individual raw spectra were baseline-subtracted and normalized using a MATLAB® processing algorithm developed in-house. For single-cell Raman imaging, the 532 nm excitation laser was raster-scanned across a 50 × 50 micron area with a step-size of 0.5 microns, yielding spectral datasets consisting of 10,000 spectra per cell. Exploiting the dramatic spectral differences arising from fluorescence of different CFZ forms, the un-processed Raman spectra were linearly deconvoluted via WiTec ProjectFOUR software’s basis component analysis using representative un-processed reference spectra obtained from untreated cells, pure free base CFZ crystals, pure CFZ-H^+^Cl^−^ crystals, and the silicon substrate. Dataset acquisition, processing, and image display parameters were performed equivalently for each cell specimen.

### CLDI injection and stabilization assay

To determine how macrophages stabilize CLDIs, mice were treated with either liposomal PBS or liposomal clodronate, as previously described^25^. 48 hours after liposome administration, mice were injected I.P. with 200 µg of CLDIs suspended in 1 mL of PBS (n = 3 mice per group per time point). At time points ranging from 0 to 48 hours, the mice were euthanized, the peritoneal lavage was collected and pelleted, and the drug content within the pellet was analyzed using the previously described spectrophotometric analysis method. Using a simple exponential regression with recovered drug content, the half-life was estimated.

### CLDI loading within individual macrophages

Using the total recovered mass of CLDIs within the liver and spleen, the CLDI loading within individual xenobiotic-sequestering macrophages was estimated using literature reported values for macrophage numbers^26^ within the liver and spleen, corrected for the percentage of cells which contained a CLDI. For the clodronate depleted organs, the reduction was accounted for with the quantified reduction in total F4/80 signal.

### Cell culture and pharmacological treatment

For pharmacological inhibition experiments, macrophage-derived RAW264.7 cells (ATCC, Manassas, VA, ATCC Number: TIB-71^TM^), at a very high seeding density of 50,000 cells/well grown in a 96 well tissue culture plate in 280 µl/well of DMEM + 10% FBS + 1% P/S (growth media), were pre-incubated (4 h) with varying concentrations of Bafilomycin A1 (BafA1; Sigma-Aldrich, St. Louis, MO, Cat. No. B1793) (0–10 nM) and NPPB (Sigma-Aldrich, St. Louis, MO, Cat. No. N4779) (0–200 µM), after which CFZ (dissolved in DMSO) was added to achieve a final concentration range of 0–10 µM. The effect of the treatment on cell viability was assessed by an XTT assay (Roche, UK).

### Drug uptake measurements

Measurement of CFZ uptake by RAW264.7 cells was performed using a modified absorbance spectroscopy method using 9 M H_2_SO_4_ (pH ≪ 0.1) to digest the entire cell population and extract CFZ from the cells. At two time points (2 and 4 h) after CFZ exposure, growth media was aspirated from the culture dish and the cells were washed twice with PBS before adding 9 M H_2_SO_4_ (100 µl/well). The plate was incubated at room temperature for 30 min before spectrophotometric quantification of CFZ using a Synergy 2 plate reader (BioTek, Winooski, VT) at 540 nm (Abs_540_) and 750 nm (Abs_750_) (for background). Standards were prepared on the same plate by adding 100 µl/well pre-determined standards of CFZ. Total CFZ uptake is reported as total intracellular drug (in picomoles) as measured and calculated using the standard curve. Assuming that the doubling time of 50,000 cells/well is ~11 h^27^, and that no compromise of cell viability occurs because of the optimum dose used for both CFZ and the pharmacological inhibitors, the total number of cells/well at the time of cellular drug uptake measurement is ~68,000.

### Experimental lysosomal pH measurements

Lysosomal pH was measured fluorometrically in peritoneal macrophages and macrophage-derived RAW264.7 cells, using methods described previously^28^. In brief, macrophages were incubated for 40 h with CFZ crystals. 20 h prior to measuring pH, cells were incubated for 15–18 h with 150 µg/ml Oregon Green-labeled dextran-10 kD (OGDx; Thermo-Fisher Scientific), and transferred to unlabeled culture medium for 3–5 h to allow OGDx to traffic fully to lysosomes. Following transfer to Ringer’s buffer, cells on Mat-tek dishes were observed in a Nikon TE300 microscope equipped for multichannel fluorescence microscopy with a 60X objective lens (N.A. 1.4). Phase contrast and three fluorescence images (excitation/emission: 440 nm/535 nm; 485 nm/535 nm; and 580 nm/630 nm) were collected from each field. The 580 nm/630 nm image was used to quantify clofazamine fluorescence. The ratio of the 485 nm/525 nm to 440 nm/525 nm images were used to measure lysosomal pH. Calibration of fluorescence ratios was obtained from cells incubated in ionophores and buffers as described in Davis and Swanson^28^. Average lysosomal pH was determined for individual cells.

### Modeling lysosomal CFZ-H^+^Cl^−^ accumulation

A Lysosomal ion regulation model which incorporates lysosomal membrane proteins, such as V-ATPase, CLC7, and membrane proton permeability was utilized^16^, and further elaborated and modified (see Supplementary Methods online) to explore the relationship between lysosomal ion homeostasis and lysosomal CFZ-H^+^Cl^−^ accumulation and stabilization. In order to incorporate the lysosomal accumulation of CFZ-H^+^Cl^−^ into the model as a function of lysosomal proton and chloride ions, the rate of overall drug accumulation obtained from experimental findings^4,29^ was used to define the rates of both lysosomal proton (H_sequestered_) and chloride (Cl_sequestered_) sequestrations by CFZ, in units of molecules per second. We assumed these rates to be equal due to the proposed equal contribution of both proton and chloride ions in the intracellular formation and accumulation of CFZ-H^+^Cl^−^ salt precipitates:

18 $${{\rm{CFZH}}}^{+}{{\rm{Cl}}}^{-}={{\rm{H}}}_{\mathrm{sequestered}}={{\rm{Cl}}}_{\mathrm{sequestered}}$$

### Model parameterization

The model incorporated 23 parameters: four were adjustable and the remaining 19 were fixed (see Supplementary Table S1). Fixed parameters were given values obtained from the literature^16,30–34^, and are associated with physiological lysosomal ion homeostasis. Therefore, these parametric values are interchangeably referred hereon as “baseline input values” or “physiological baseline input values”. The adjustable parameters are those values that were varied from their respective baseline input values in order to investigate their individual as well as combined effects on the physiological lysosomal pH, Cl^−^, and membrane potential readout values, as discussed in the following subsections. These parameters include the number of active V-ATPase and CLC7 molecules per lysosome, as well as the cytoplasmic chloride concentration. In addition, the rates of proton and chloride sequestrations by CFZ are considered adjustable parameters as they are foreign to the lysosome.

### Simulating lysosomal CFZ-H^+^Cl^−^ accumulation

The previously mentioned rates of proton and chloride sequestrations by CFZ were adjusted to either 0.01 or 0.1 picomoles/cell/day^4,29^. However, in our model simulations, we converted the units of these rates to picomoles/lysosome/day assuming there are ~100 lysosomes in a cell. From here onwards, we interchangeably refer to these rates, 0.01 and 0.1 picomoles/cell/day, as 1-fold (1X) and 10-fold (10X) rates of lysosomal CFZ-H^+^Cl^−^ accumulation, respectively.

### Simulating changes in lysosomal membrane proteins and cytoplasmic chloride amounts

In order to study the roles of V-ATPase, CLC7, and cytoplasmic chloride concentration on lysosomal accumulation of CFZ-H^+^Cl^−^, we varied the total numbers of active V-ATPase and CLC7 molecules per lysosome, and the cytoplasmic chloride concentration, while fixing the values of other lysosomal parameters at their baseline physiological input values in the presence as well as absence of lysosomal CFZ-H^+^Cl^−^ accumulation. More specifically, to simulate the simultaneous inhibition of V-ATPase and CLC7, the total number of CLC7 molecules per lysosome was varied from 0 to 5,000 (resulting in 7–16 data-generating points) while, one parametric simulation at a time, the total number of V-ATPase molecules per lysosome was manually varied from 0 to 300 (resulting in 4–7 data-generating points). Moreover, to simulate the simultaneous inhibition of V-ATPase and cytoplasmic chloride, the cytoplasmic chloride concentration was varied from 0 to 10 mM (resulting in 16 data-generating points) while, one parametric simulation at a time, the total number of V-ATPase molecules per lysosome was manually varied from 0 to 300 (resulting in 4–7 data-generating points).

Using the aforementioned ranges of the adjustable lysosomal parameters, the corresponding lysosomal parameter inhibition range of 0 to 100% was calculated; where 0% represents no change from respective physiological baseline input value, and 100% represents the input value set to ~zero. The inhibition range was calculated as follows by comparing the corresponding input value (Adjusted Input Value) from the aforementioned given range with its respective physiological input value (Baseline Input Value):

19 $$ \% \,{\rm{Inhibition}}=\frac{{\rm{Baseline}}\,{\rm{Input}}\,{\rm{Value}}-{\rm{Adjusted}}\,{\rm{Input}}\,\mathrm{Value}\,}{{\rm{Baseline}}\,{\rm{Input}}\,{\rm{Value}}}\times 100 \% $$

### Calculating the effect of lysosomal CFZ-H^+^Cl^−^ accumulation on lysosomal ion homeostasis

Final lysosomal pH, chloride, and membrane potential were chosen as readout values because they are direct indicators of lysosomal ion homeostasis and physiology. Thus, parametric simulations of these variables were performed as a function of the aforementioned ranges of V-ATPase, CLC7, and cytoplasmic chloride in the absence and presence of lysosomal CFZ-H^+^Cl^−^ accumulation at the rate of either 1X or 10X. Then, the readout values in the absence of lysosomal CFZ-H^+^Cl^−^ accumulation were subtracted from those in the presence of lysosomal CFZ-H^+^Cl^−^ accumulation in order to determine the effect of lysosomal CFZ-H^+^Cl^−^ accumulation on lysosomal ion homeostasis.

### Calculating the effects of V-ATPase, chloride channels, and cytoplasmic chloride on the physiological lysosomal accumulation of CFZ-H^+^Cl^−^

Time-plot simulations, where all of the lysosomal parameters were set to their respective baseline values in the absence of lysosomal CFZ-H^+^Cl^−^ accumulation, were performed to obtain physiological final lysosomal pH, chloride accumulation, and membrane potential values, which from hereon we refer to as “physiological baseline readout values”. Moreover, model parametric simulations of final lysosomal pH, Cl^−^, and membrane potential as a function of simultaneous V-ATPase-CLC7 and V-ATPase-cytoplasmic chloride inhibitions were performed in the presence of lysosomal CFZ-H^+^Cl^−^ accumulation at the rates of 1X and 10X. These were then subtracted from the physiological baseline readout values in order to calculate the effects of the simultaneous lysosomal parameter inhibitions on the dose-dependent lysosomal CFZ-H^+^Cl^−^ accumulation.

### Confirmation of steady-state and mass balance

For all of the aforementioned simulations, the final readout values were confirmed that they were steady-state values by performing the simulations for >24 h. We confirmed that mass balance was maintained in all of the simulations in the presence and absence of lysosomal CFZ-H^+^Cl^−^ accumulation as long as a physiological pH gradient of up to 4.6 pH units was maintained across the lysosomal membrane.

## Results

### The weakly basic building block has pH-dependent solubility properties

**Figure 1:**
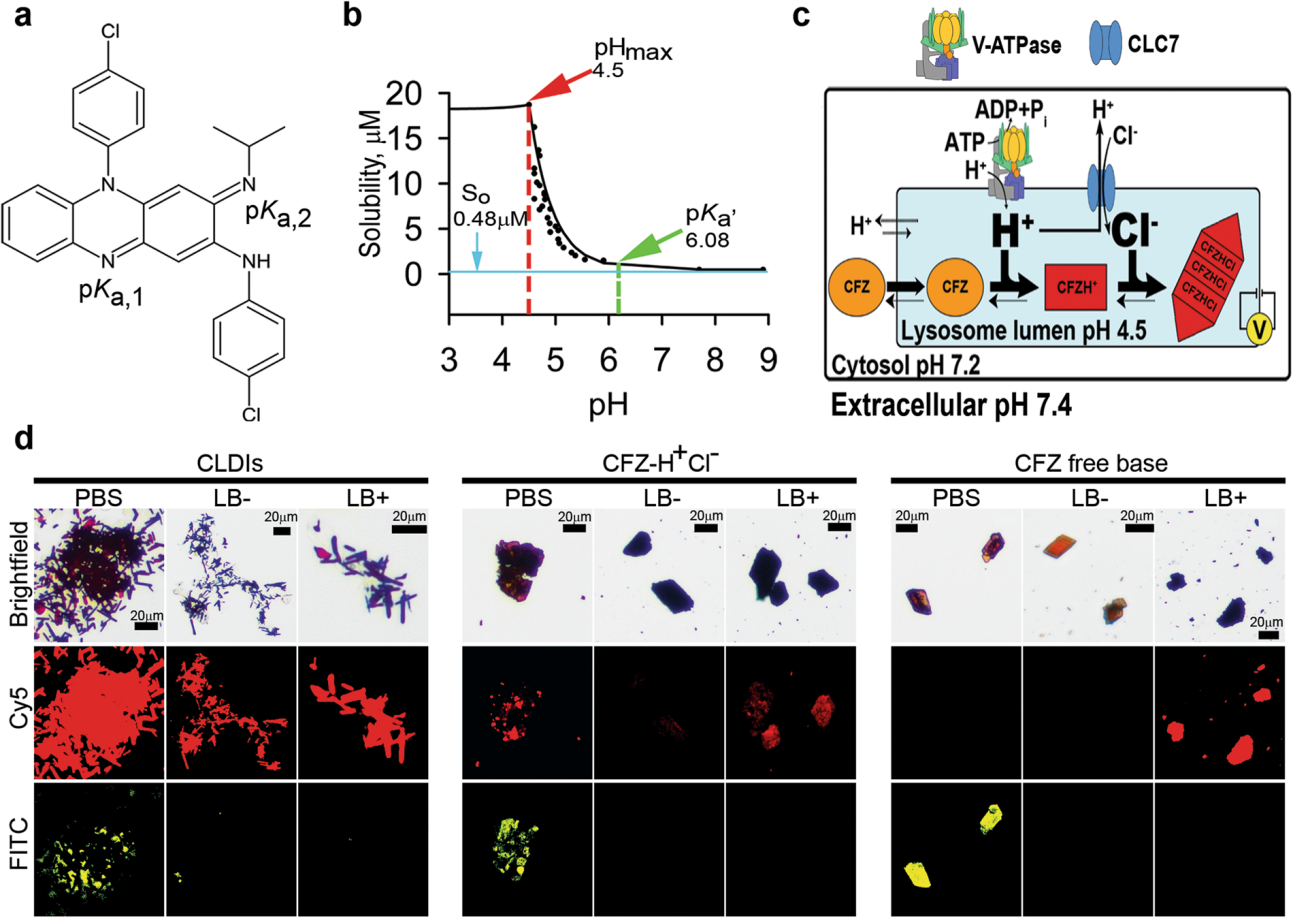
Chemical characterization of CFZ. (**a**) Chemical structure of clofazimine (CFZ) with its two protonation sites and corresponding predicted (chemi-informatic) p*K*_a_ values (p*K*_a,1_ = 2.31 and p*K*_a,2_ = 9.29). (**b**) CFZ-H^+^Cl^−^ solubility-pH study revealed the solution pH dependence of the stabilization of the free base versus salt form of the drug with respect to its solubility parameters; which include the intrinsic free base solubility (S_o_), apparent p*K*_a,2_ (p*K*_a_’), and pH_max_. (**c**) Illustration showing the cellular and subcellular accumulation of free base CFZ, its subsequent protonation (CFZH^+^), and ion-ion interaction of CFZH^+^ and cellular Cl^−^ to form CFZ-H^+^Cl^−^. This phenomenon depends on the drug’s intrinsic solubility properties as well as the cellular pH and Cl^−^ levels, which are primarily regulated by membrane proteins: proton-pump known as V-ATPase and Cl^−^/H^+^ antiporter known as CLC7. (**d**) Stability of CLDIs, CFZ-H^+^Cl^−^, and free base CFZ in PBS (pH 7.4), lysosomal buffer without sodium chloride (LB−, pH 4.5), and lysosomal buffer with 100 mM sodium chloride (LB+, pH 4.5) was monitored via brightfield and fluorescence microscopy.

CFZ is a weak base with two amines, which can be protonated depending on the pH of the immediate environment (Fig. 1a). To study its pH-dependent solubility behavior, we used an established approach^19^ (see Supplementary Methods online for detailed explanation), which allowed an accurate measurement of the total solubility of CFZ-H^+^Cl^−^ as a function of pH. From the experimental measurements, we calculated the solubility properties of CFZ, in an aqueous media at 25 °C, which include its apparent p*K*_a,2_ (p*K*_a’_ = 6.08 ± 2.43 × 10^−3^; 95% Confidence Interval = 6.07, 6.09) and intrinsic free base solubility (S_0_ = 0.48 ± 4.05 × 10^−6^ µM; 95% CI = 0.48, 0.48) (Fig. 1b). Moreover, to determine the effect of lysosomal Cl^−^ (Fig. 1c) on the stability of the free base versus salt form of the drug, we performed drug stability experiments (Fig. 1d) in the presence and absence of lysosomal buffer (pH 4.5, Cl^−^ concentration = 0–100 mM). We monitored drug stability by relying on the unique fluorescence profile of the different forms of CFZ: CFZ free base exhibits green and red fluorescence; peak excitation: 540–560 nm, peak emission: 560–600 nm, whereas CFZ-H^+^Cl^−^ exhibits red and far red fluorescence; peak excitation: 560–600 nm, peak emission: 650–690 nm^11^. Indeed, the fluorescence of CLDIs resembles the fluorescence of the synthesized CFZ-H^+^Cl^−^ crystals (peak excitation: 560–600 nm, peak emission: 650–690 nm) (see Supplementary Fig. S1).

A low pH environment in the absence of Cl^−^ (LB−) induced the destabilization of both CFZ-H^+^Cl^−^ and free base CFZ, without affecting the stability of CLDIs. To the contrary, a low pH environment in the presence of a physiological lysosomal Cl^−^ (LB+) induced the stabilization of CFZ-H^+^Cl^−^ and CLDIs, whereas the free base CFZ precipitated out as CFZ-H^+^Cl^−^ (Fig. 1d). Thus, these findings reveal that the precipitation of CFZ as a hydrochloride salt was highly sensitive to small, but physiologically relevant variations in pH and chloride concentrations (pH_max_ = 4.5 ± 7.11 × 10^−15^, 95% CI = 4.5, 4.5; and *K*_sp_ = 332.3 ± 3.71 µM^2^, 95% CI = 323.1, 341.5) (Fig. 1b,d); where the pH_max_ reveals the pH below which CFZ-H^+^Cl^−^ salt formation occurs, and the *K*_sp_ elucidates the Cl^−^ concentration, which is >(*K*_sp_)^1/2^ µM, that would further stabilize the salt form of the drug through a common-ion effect^35^. Thus, at the pharmacologically-relevant CFZ concentrations that have previously been measured in serum (1 to 20 µM), local variations in pH (~7.4 extracellular; ~7.2 cytosolic, and ~4.5 lysosomal) and chloride concentrations (~80 mM extracellular; ~10–20 mM in cytosol; and ~50–200 mM in lysosomes) can lead to a greater propensity of CFZ to precipitate as a hydrochloride salt in the lysosomal microenvironment (Fig. 1c,d).

### The intracellular organization of the self-assembled mechanopharmaceutical device

**Figure 2:**
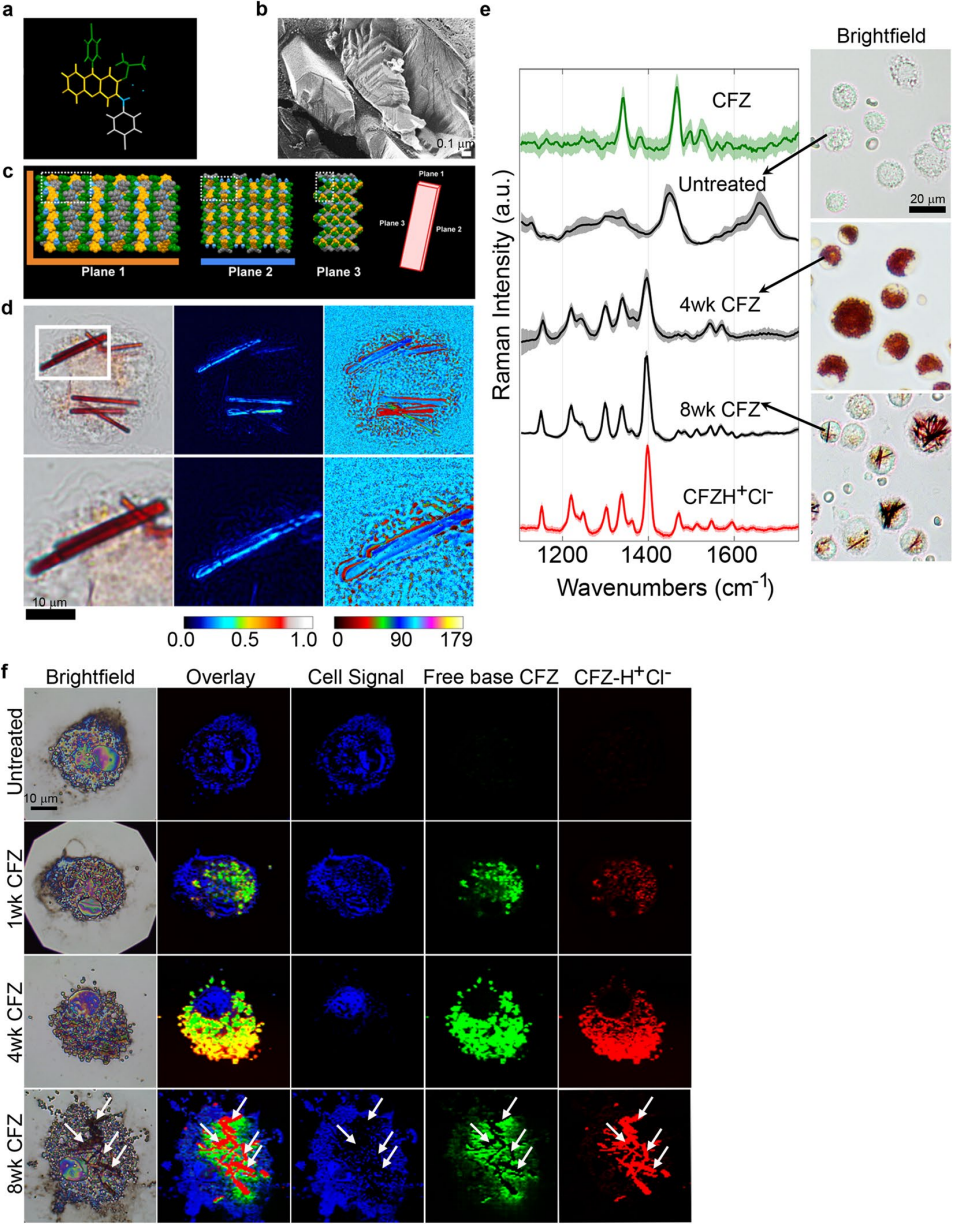
Intracellular self-assembled crystal organization of CFZ-H^+^Cl^−^. (**a**) CFZ-H^+^Cl^−^ chemical structure with color coding corresponding to the regions of the structure in the crystal arrangement. (**b**) Freeze-fracture electron micrograph of CLDIs within a Kupffer cell, showing that the CLDIs are sequestered inside the cell as multiple layered planes with lamellar spacing of 6 nm to 14 nm, by a double membrane of biological origin (~20 nm in thickness)^21^. (**c)** Crystal packing of CFZ-H^+^Cl^−^ showing the three crystallographic planes of the crystal: a hydrophobic face in orange (plane 1), a hydrophilic face in blue (plane 2), and an amphipathic face (plane 3)^8^. (**d**) CLDIs (100–1.5X magnification) zoomed in (zoom factor of 2) in alveolar macrophages show high dichroism and an axis of highest transmittance, with a different profile (represented by the different signal intensity associated with the color bar) observed specifically around the edges of the crystal. (**e**) Raman spectra of free base CFZ, CFZ-H^+^Cl^−^, untreated and treated alveolar macrophages (4 and 8wks CFZ-fed mice) show distinct Raman peaks distinguishing between the different forms of the drug (red in the brightfield images): free base CFZ (1341 and 1465 cm^−1^), CFZ-H^+^Cl^−^ (1399 cm^−1^). (**f)** Reflected brightfield and Raman images of alveolar macrophages from CFZ-fed mice prepared on silicon chip substrates; basis spectral fitting was used to show the temporal accumulation and cytoplasmic packaging of CFZ into highly ordered CLDIs.

Prolonged administration of CFZ results in its accumulation in both human and animals as microscopic, insoluble, and membrane-bound aggregates, known as Crystal Like Drug Inclusions (CLDIs), which are primarily found within tissue macrophages^3–5^. CLDIs are primarily comprised of a protonated, hydrochloride salt of CFZ (Fig. 2a)^7^, which forms faceted structures possessing three orthogonal cleavage and multiple fracture planes with lamellar spacing of 6 nm to 14 nm^21^ (Fig. 2b). These orthogonal cleavage and fracture planes correspond to the orthogonal crystallographic planes of the CFZ-H^+^Cl^−^ unit cell (Fig. 2c). When examined under polarized light (623 nm wavelength), the orthorhombic crystal structure of the deep red CLDIs^11,29,36^ exhibits dichroism (Fig. 2d)^24^. Due to the biological origin of the CLDIs^7^, the interaction between the cell and crystal often leads to significant variations in its organization, impacting the manner in which it interacts with polarized light (Fig. 2d). Furthermore, reference spectra of pure CFZ and CFZ-H^+^Cl^−^ identified characteristic Raman peaks for clear distinction between the two forms (Fig. 2e). Spectra obtained from cytoplasmic regions of untreated mouse alveolar macrophages were typical of biological specimens with no apparent contributions from drug. However, spectra obtained from cytoplasmic regions of 4- and 8-week CFZ-fed mouse alveolar macrophages revealed the presence of CFZ-H^+^Cl^−^, which dominated any Raman signal associated with biological specimens, and exhibited unique peak intensities and peak widths, which attribute to the geometric organization of the supramolecular structures: peaks generally become narrower and more intense with higher degree of order in samples, which suggests that by 8 weeks, the macrophages have organized the accumulated drug into supramolecular crystalline packages of CFZ-H^+^Cl^−^ salt. Moreover, Raman imaging of single-cells revealed cytoplasmic accumulation of both forms of the drug after just 1 week and continuing through 4 weeks; at 8 weeks CLDI formation was apparent and clearly distinguishable from free base CFZ, as evidenced by non-overlapping Raman signals in the biocrystal regions of Raman image (Fig. 2f).

### Macrophages are necessary for the self-assembly of the mechanopharmaceutical device

**Figure 3:**
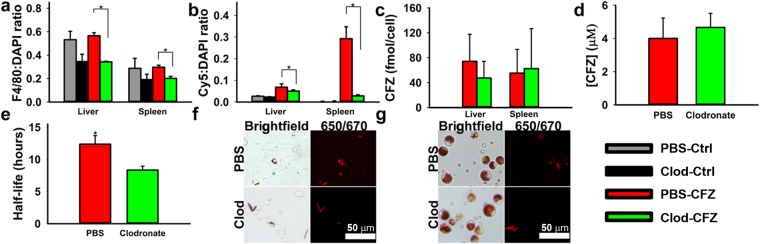
The role of macrophages on the intracellular accumulation of CFZ biocrystals *in vivo*. (**a**) Clodronate depletion significantly reduces both hepatic and splenic macrophages in Clodronate-CFZ treatment group. (**b**) Depletion of macrophages results in concomitant reduction in CLDI loading within the livers and spleens of treated animals. (**c**) Clodronate depletion does not significantly reduce the estimated clofazimine loading per macrophage in the livers and spleen. (**d**) Clodronate depletion does not significantly impact the serum concentrations of clofazimine following four weeks of treatment. (**e**) Depletion of peritoneal macrophages significantly increases the rate of degradation of CLDIs injected intraperitoneally, leading to a significantly faster half-life. (**f**) Peritoneal exudate of PBS- and clodronate-liposome treated mice 48 hours following CLDI injection showing brightfield and Cy5 fluorescence (red fluorescence and red in brightfield images correspond to CLDIs) (*p < 0.05, ANOVA or ANOVA, Tukey’s HSD when applicable). (**g**) Alveolar lavage from PBS- and clodronate-liposome treated mice. Clodronate treatment did not impact CLDI accumulation (red fluorescence and red in brightfield images correspond to CLDIs) within alveolar macrophages.

To investigate whether macrophages play an active role in stabilizing CLDIs *in vivo*, macrophages were depleted by injecting mice (n = 3–4 per treatment group) intraperitoneally with clodronate liposomes. This treatment depletes phagocytic cells without affecting non-phagocytic cells^20^. Moreover, the aforementioned CFZ’s unique fluorescence characteristics were used to monitor the effect of macrophage depletion on the accumulation of CFZ-H^+^Cl^−^ crystal. Following liposome therapy, the relative number of F4/80 positive macrophages within the liver and spleen of each treatment group was quantified using the total ratio of F4/80 to nuclear signal, revealing a significant reduction in the total macrophage population in the clodronate-CFZ fed group when compared to the PBS-CFZ treated group (p < 0.05, ANOVA, Tukey’s HSD) (Fig. 3a). Consequently, using the ratio between the intrinsic far-red fluorescence of CFZ-H^+^Cl^−^ ^37^ and total nuclear signal, there was a significant reduction in the accumulation and stabilization of CFZ-H^+^Cl^−^ within the liver and spleen (p < 0.05, ANOVA, Tukey’s HSD) compared to the PBS-CFZ treated group (Fig. 3b). More specifically, within the PBS-CFZ treated mice, the livers accumulated an average of 0.68 ± 0.27 mg CFZ/g tissue, while the clodronate-CFZ livers accumulated an average of 0.23 ± 0.09 mg CFZ/g tissue (p < 0.05, two-tailed Student’s t-test); a reduction of nearly 66%. Similarly, the spleens of PBS-CFZ treated mice accumulated an average of 2.31 ± 0.40 mg CFZ/g tissue, compared to 0.70 ± 0.12 mg CFZ/g tissue in the clodronate-CFZ groups (p < 0.01, two-tailed Student’s t-test); a reduction of 70%.

The individual drug loading within the macrophages of the liver and spleen was estimated based off of literature-reported values for macrophage populations in those two organs^26^ and the total recovered drug within each organ, and there was no difference in total drug loading within either population of macrophage as a result of clodronate therapy (Fig. 3c). Furthermore, there was no significant difference in mean plasma concentrations of CFZ at the end of therapy (Fig. 3d). Taken together, this provides evidence that, while clodronate therapy was reducing the macrophage population and subsequent bioaccumulation within the liver and spleen, it was not impacting the general bioavailability and absorption of CFZ in the body. To determine how macrophages stabilized the self-assembled mechanopharmaceutical devices, isolated CLDIs were injected I.P. into clodronate and PBS liposome treated mice and collected at time points from 0 to 48 hours (n = 3 mice per group per time point). Without macrophages present in the peritoneal cavity to internalize the CLDI, there was a significant reduction in the estimated half-life of the biocrystal (p < 0.05, ANOVA) (Fig. 3e). This was confirmed via microscopic analysis of the peritoneal exudate 48 hours post-injection, with CLDIs becoming internalized by the macrophage and retaining their far-red fluorescence in the PBS-treatment group, while the clodronate-treated group showed reduced CLDI content within the exudate (Fig. 3f).

Additionally, in order to confirm that clodronate did not impact drug accumulation within other macrophage populations, alveolar macrophages were collected and analyzed. Within the alveolar macrophage population, there was no significant difference in total cell number collected between the PBS and clodronate-treated groups (p = 0.48, two-tailed Student’s t-test), as well as in the ability of the cells to accumulate CFZ in the form of a CLDI; where each alveolar macrophage population contained approximately 20% CLDI-positive cells (p = 0.36, two-tailed Student’s t-test) (Fig. 3g). Thus, the specific effect of clodronate liposomes on liver and spleen CLDIs and CFZ content, as well as the increased rate of CLDI destabilization and solubilization in the absence of peritoneal macrophages provide evidence that these cells are directly responsible for the *in vivo* accumulation, self-assembly, and stabilization of CFZ-H^+^Cl^−^.

### The intracellular accumulation, protonation, and self-assembly of the building block in cytoplasmic vesicles does not interfere with lysosomal pH

**Figure 4:**
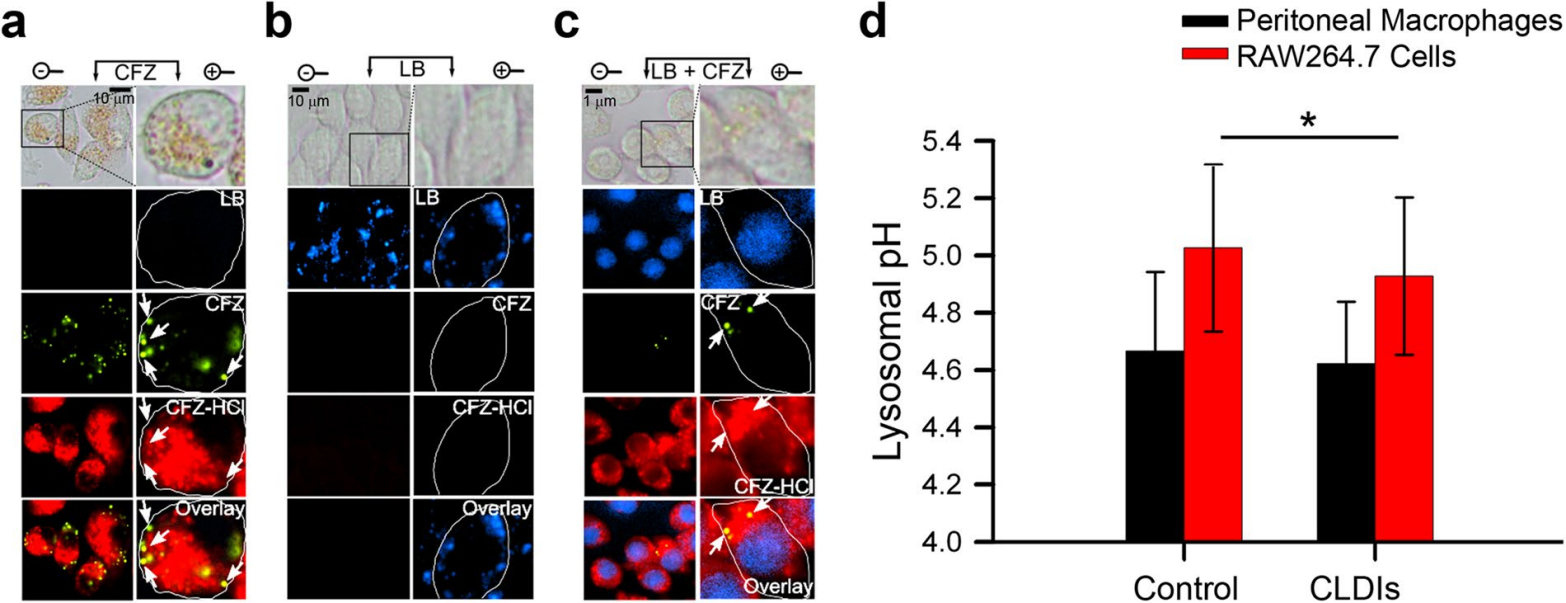
Evidence of lysosomal accumulation of CFZ via fluorescence staining patterns in RAW264.7 cells. (**a**) Epifluorescence microscopy of RAW264.7 cells (40X magnification) zoomed in (zoom factor of 2.5) when incubated with (**a**) CFZ (10 μM), (**b**) Lysotracker Blue (LB, 1 μM), and (**c**) CFZ (10 μM) and LB (1 μM) together at *t* = 24 hours. Green fluorescence spots indicative of CFZ are also further annotated using white arrows for cross-referencing with other images. Note the lack of any far-red fluorescence positive signal in the direction pointed by the arrows. Vesicular staining pattern of LB visible as blue punctate spots in (**b)** are absent in (**c**), while nuclear staining becomes visible due to LB displacement from the lysosome to the nucleus because of CFZ accumulation in the lysosome. Cell boundaries are shown in white in the digitally zoomed panels. (**d**) Lysosomal pH measurements of RAW26.7 cells in the presence and absence of CLDIs, and peritoneal macrophages of control versus 8-week CFZ-fed mice. Data are mean ± S.D. of 29–181 measurements; *p = 0.026; analyzed by unpaired two-tailed Student *t*-test.

Taking advantage of the aforementioned CFZ’s unique fluorescence properties, we studied the accumulation of the un-protonated free base and protonated hydrochloride salt form of the drug in macrophages. Incubation of macrophages with CFZ *in vitro* revealed vesicular staining pattern consistent with CFZ free base (peak excitation: 540–560 nm, peak emission: 560–600 nm) as well as CFZ-H^+^Cl^−^ (peak excitation: 560–600 nm, peak emission: 650–690 nm)^11^. Both fluorescent forms were visible in CFZ-treated cells and absent in untreated, control cells (Fig. 4a and see Supplementary Fig. S2). Based on the observed little overlap of their orthogonal fluorescence signals, CFZ-H^+^Cl^−^ did not co-localize with free base CFZ (Pearson’s Colocalization Coefficient - PCC between green and far-red fluorescence = 0.30 ± 0.29) (see Supplementary Fig. S3). In addition, when cells were co-incubated with both CFZ and Lysotracker Blue (LB), LB fluorescence signal (excitation: 373 nm, emission: 422 nm) was inhibited in the cytoplasm and displaced^38^ to the nucleus (based on the observed nuclear staining), whereas the punctate green and far-red fluorescence of CFZ were clearly visible in the cytoplasm of these cells (Fig. 4c, and see Supplementary Figs S2 and S3). As a control experiment, we measured the lysosomal pH of macrophages following phagocytosis of CLDIs in macrophage-derived RAW264.7 cells as well as in peritoneal macrophages of 8-week CFZ-fed mice (Fig. 4d). In both cases, the lysosomal pH was not physiologically affected by the presence of the CFZ, as reflected by <0.1 pH unit difference between the drug-free and drug-containing peritoneal macrophages and RAW264.7 cells. Thus, CFZ uptake in macrophage lysosomes does not perturb physiological lysosomal pH homeostasis, which is required for various lysosomal functions, such as lysosomal trafficking and degradation of intrinsic and extrinsic materials within the lysosome by various pH-dependent hydrolytic lysosomal enzymes, which are essential for overall cellular homeostasis and viability^39^. Moreover, because the fluorescence signals of Lysotracker® probes are pH-independent^40^, we interpret this to mean that intralysosomal CFZ accumulation interferes with Lysotracker probe accumulation through a mechanism that is independent of changes in lysosomal pH.

### The building block’s self-assembly mechanism is biocompatible with the lysosome

**Figure 5:**
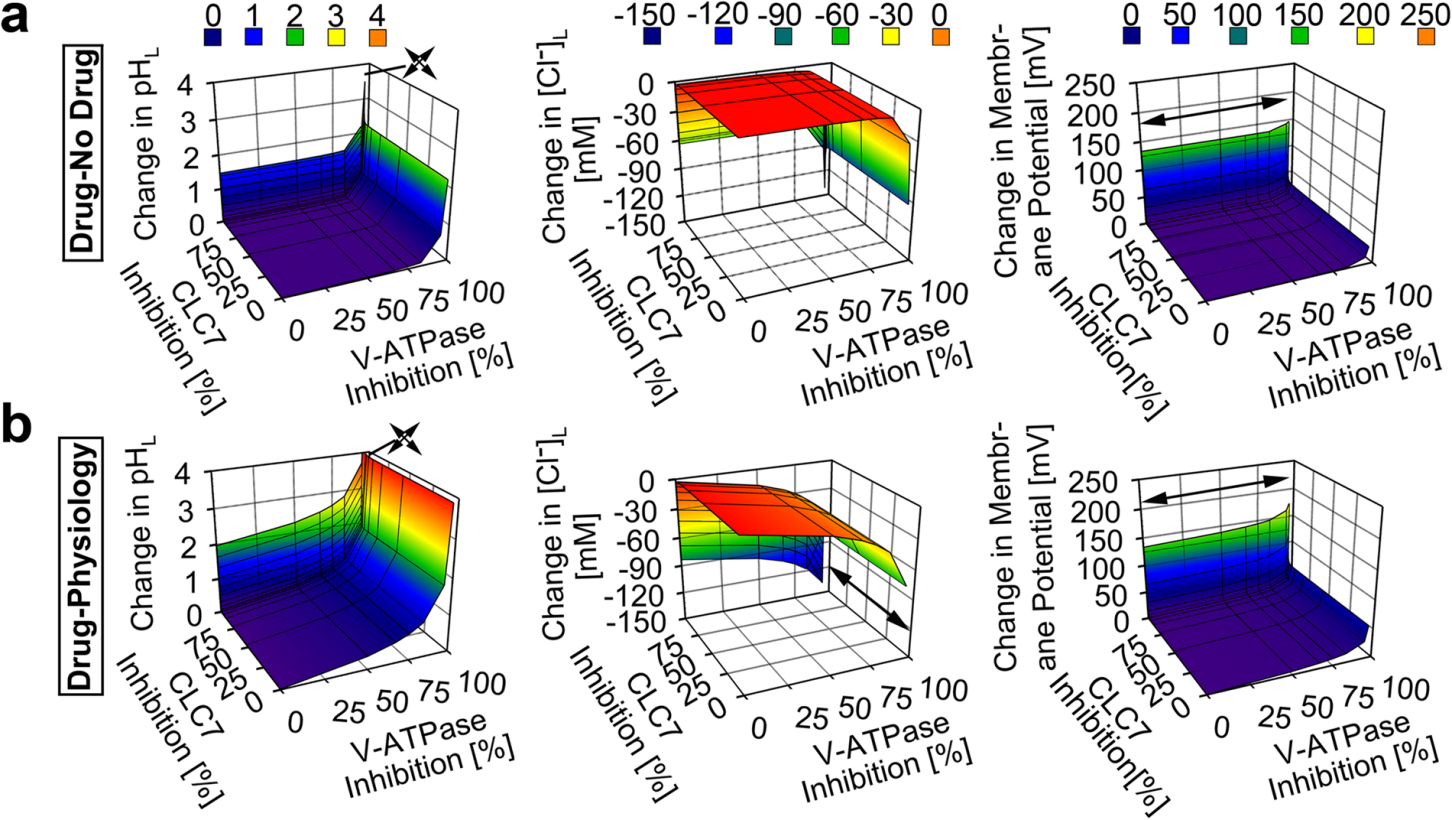
Model and simulation of the effects of V-ATPase and CLC7 on the lysosomal accumulation of CFZ-H^+^Cl^−^. (**a**) V-ATPase inhibition showed a more substantial effect than CLC7 inhibition on the accumulation of CFZ-H^+^Cl^−^ at the rate of 0.01 picomol/cell/day, as reflected by the changes in the lysosomal pH, Cl^−^, and membrane potential values of the CFZ-H^+^Cl^−^ containing lysosome from that of the CFZ-H^+^Cl^−^ free lysosome. (**b**) V-ATPase inhibition showed a more substantial effect than CLC7 inhibition on the physiological accumulation of CFZ-H^+^Cl^−^ at the rate of 0.01 picomol/cell/day, as reflected by the changes in the lysosomal pH, Cl^−^, and membrane potential values of the CFZ-H^+^Cl^−^ containing lysosome from respective baseline physiological values. Arrow signs represent values outside of the axes plot range.

To identify the candidate mechanism driving CFZ-H^+^Cl^−^ accumulation and stabilization in lysosomes, we used a well-established systems-based mathematical model of lysosomal ion regulation. We first performed a simultaneous variation of the number of active V-ATPase and chloride channels per lysosome (resulting in 0 to 100% inhibition of both parameters from their respective baseline physiological input values of 300 and 5000) in the presence and absence of dose-dependent lysosomal CFZ-H^+^Cl^−^ accumulation (0 to 0.01 picomoles/cell/day), as measured *in vitro*^4,29^. The simulation results indicated that CFZ-H^+^Cl^−^ accumulates at the measured rate of 0.01 picomoles/cell/day exerting a negligible effect on lysosomal pH, Cl^−^, and membrane potential from respective physiological baseline readout values (4.53 pH units, 224.6 mM, and 0.79 mV). Only a significant (≥97%) reduction in the total V-ATPase amount per lysosome led to changes in these values (Fig. 5a). Consistent with this, <97% V-ATPase inhibition per lysosome was required for the physiological accommodation of lysosomal CFZ-H^+^Cl^−^ accumulating at 0.01 picomoles/cell/day (Fig. 5b). However, unlike the number of V-ATPase, the number of chloride channel molecules per lysosome did not show any effect on the physiological CFZ-H^+^Cl^−^ accumulation unless this chloride current was completely absent (Fig. 5b).

**Figure 6:**
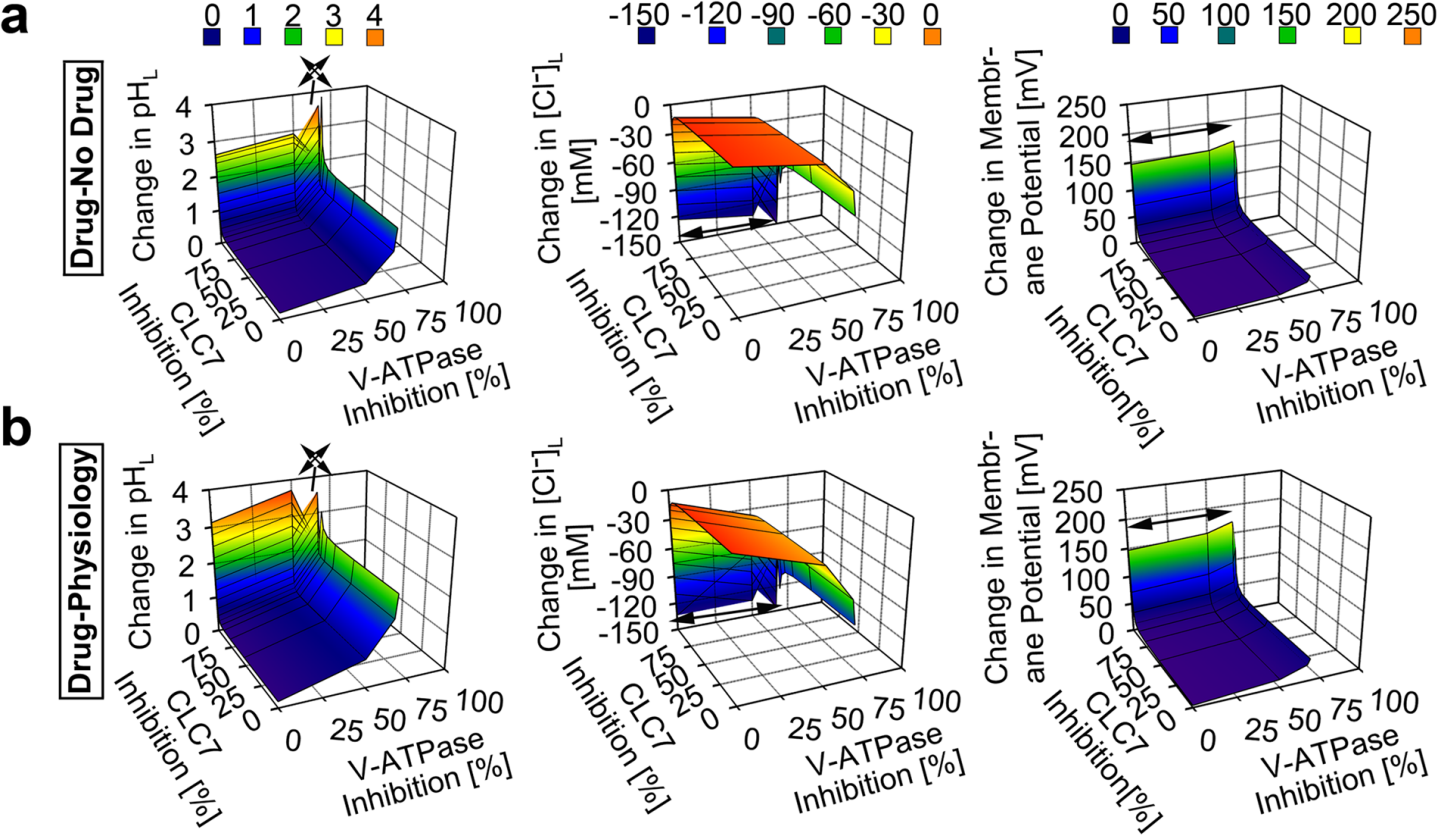
Model and simulation of the effects of V-ATPase and CLC7 on the lysosomal accumulation of CFZ-H^+^Cl^−^ at a higher dose. (**a**) V-ATPase inhibition showed a more substantial effect than CLC7 inhibition on the accumulation of CFZ-H^+^Cl^−^ at the rate of 0.1 picomol/cell/day, as reflected by the changes in the lysosomal pH, Cl^−^, and membrane potential values of the CFZ-H^+^Cl^−^ containing lysosome from that of the CFZ-H^+^Cl^−^ free lysosome. (**b**) V-ATPase inhibition showed a more substantial effect than CLC7 inhibition on the physiological accumulation of CFZ-H^+^Cl^−^ at the rate of 0.1 picomol/cell/day, as reflected by the changes in the lysosomal pH, Cl^−^, and membrane potential values of the CFZ-H^+^Cl^−^ containing lysosome from respective baseline physiological values. Arrow signs represent values outside of the axes plot range.

In dose-dependent simulations, CFZ-H^+^Cl^−^ accumulating at a ten-fold greater (non-physiological) rate of 0.1 picomoles/cell/day led to more pronounced changes in lysosomal pH, Cl^−^, and membrane potential (Fig. 6a). At least <68.3% V-ATPase inhibition per lysosome was required to sustain physiological ion concentrations in the presence of this 10-fold greater rate of CFZ-H^+^Cl^−^ accumulation (Fig. 6b). In contrast, variations in the numbers of chloride channels did not affect the physiological lysosomal accumulation of CFZ-H^+^Cl^−^ at a rate of 0.1 picomoles/cell/day, unless there was a complete inhibition of the chloride current (Fig. 6b).

### V-ATPase is essential in the building block accumulation and self-assembly

**Figure 7:**
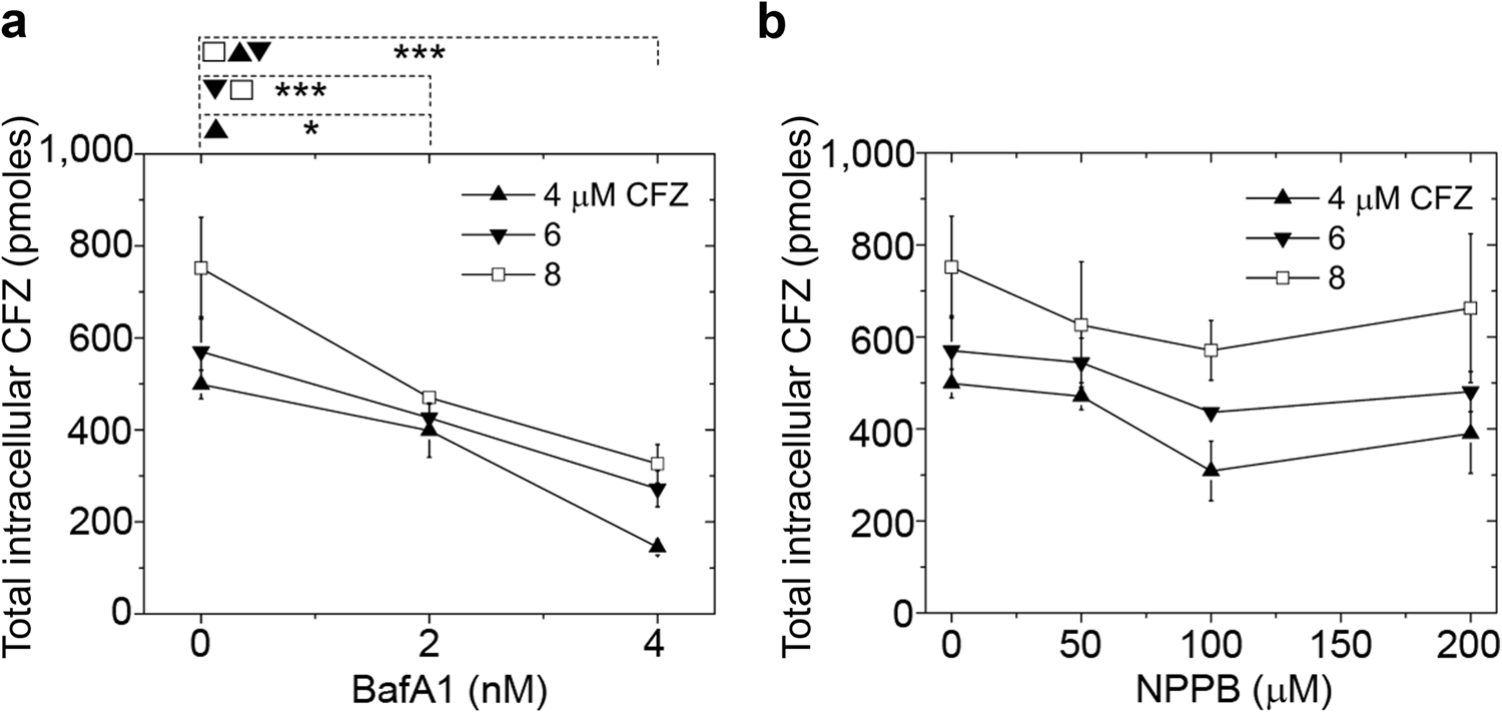
Intracellular CFZ accumulation. Total intracellular CFZ accumulation in picomoles (pmoles) in RAW264.7 cells (~68,000 cells/well) when pre-incubated with (**a**) BafA1 and (**b**) NPPB and initial concentration of CFZ = 4, 6, and 8 μM at *t* = 4 hours. Significant inhibition of CFZ accumulation was measured at *t* = 4 hours when cells were exposed to BafA1. To the contrary, no such inhibition was measured when cells were exposed to NPPB at *t* = 4 hours. Data are the mean ± S.D. of six experiments. *p < 0.05, **p < 0.01, ***p < 0.001. Each data point was compared in a pair-wise independent Student t-test.

To verify our model and simulation findings, we performed experiments to detect the effects of V-ATPase and chloride channels on the intracellular accumulation of CFZ. All experiments regarding the incubation and accumulation of CFZ were performed under conditions in which cellular viability was maintained. In the absence of any inhibitor, 8 µM extracellular CFZ accumulated at a rate of 0.01 picomoles/cell/day, as previously reported^4,29^. However, inhibiting the V-ATPase with BafA1 reduced cellular CFZ accumulation by 80 ± 13% (p < 0.01) in a 4 h incubation, relative to control, uninhibited cells (Fig. 7a). This observation is consistent with our simulation results, where up to 50% V-ATPase inhibition resulted in a lysosomal pH increment (Fig. 5a), which associates with a reduction in cellular drug accumulation. In contrast, when cells were treated with the chloride channel inhibitor NPPB and exposed to CFZ, no significant change was measured in the accumulation of CFZ following a 4 h incubation (Fig. 7b). This contrasts with the effect observed with BafA1 (Fig. 7a), and is consistent with our simulation results (Fig. 5a), thereby indicating that in the presence of CFZ uptake, lysosomal ion homeostasis is insensitive to chloride channel inhibition.

## Discussion

As a weakly basic, poorly soluble small-molecule chemical that is FDA-approved, orally bioavailable, slowly cleared, and administered at high doses for prolonged periods of time, CFZ affords us the opportunity to reverse engineer the construction of a mechanopharmaceutical device that was found to form naturally inside cells. We report that, because of its physicochemical properties, CFZ is highly prone to precipitation in acidic endolysosomal compartments in macrophages, where it self-assembles into a highly ordered CFZ-H^+^Cl^−^ biocrystal, possessing many interesting optical and biomechanical features. Indeed, the preferential accumulation of CFZ in macrophage lysosomes is evident based on the displacement of Lysotracker Blue fluorescence from the cytoplasmic vesicles (Fig. 4). Furthermore, fluorescence imaging revealed that CFZ was present both as a free base and hydrochloride salt form within intracellular vesicles (Fig. 4). Thus, we performed computational simulations and experiments to probe the relationship between CFZ’s pH-dependent solubility properties, the formation of salt and free base crystals, and the molecular, ion transport mechanisms responsible for this mechanopharmaceutical phenomenon, which could be exploited for developing other weakly basic small molecule building blocks as cell-targeted, self-assembling mechanopharmaceutical devices^41,42^.

Despite the complexities of lysosomal ion homeostasis, computational simulations and experimental results provide a straightforward explanation for the observed behavior of CFZ: the number of V-ATPase molecules that drive the lowering of macrophage lysosomal pH^43^ plays a critical role in determining the intracellular bioaccumulation of CFZ in lysosomes without affecting lysosomal ion homeostasis. Our results show that very high levels of V-ATPase expression, that are typical of macrophages, are both necessary and sufficient to explain the accumulation and stabilization of CFZ-H^+^Cl^−^ salt in these cells because they allow the cells to maintain physiological lysosomal pH and membrane potential in spite of the accumulation of the drug. In addition, the intrinsic solubility properties of CFZ are such that the protonated form of the drug strongly interacts with chloride in a concentration range that is typically observed in lysosomes, which leads to the physiological accumulation of the drug in its hydrochloride salt form. Accordingly, V-ATPase expression establishes the ability of the cell to maintain physiological lysosomal pH^44^, which, in relation to the drug’s pH_max_ and *K*_sp_ (Fig. 1), can sustain the continuous accumulation of the hydrochloride salt form of the drug^19^ in the lysosome.

More importantly, we found that experimental results^4,29^ were highly consistent with model simulation results. First, the inhibition of V-ATPase activity resulted in the reduction of the total intracellular CFZ accumulation (Fig. 7a), which was consistent with the model simulation results (Figs 5 and 6). In contrast, NPPB-mediated reduction of chloride channel activity did not affect the accumulation and stabilization of the salt form of the drug (Fig. 7b), which was also consistent with the model simulation results (Figs 5 and 6, and see Supplementary Figs S5 and S6). Furthermore, the measured *K*_sp_ of CFZ-H^+^Cl^−^ and our CLDIs/CFZ-H^+^Cl^−^ stability results (Fig. 1) indicate that the physiological lysosomal chloride levels are sufficient to stabilize CFZ-H^+^Cl^−^ salt formation through a common-ion “salting-out” mechanism^35^. Therefore, our findings collectively provide the first mechanistic framework that explains the accumulation of highly ordered, self-assembled CFZ-H^+^Cl^−^ salt biocrystals in macrophages (Figs 2 and 3); which depends on the high levels of V-ATPase expression that is unique to this cell type^16^.

It is also noteworthy that macrophages have lysosomes which are an order of magnitude greater in size than that of other cell types^45^. This provides them with the capability to accommodate massive accumulation of weakly basic drugs^46^. Even in cases where the initial lysosomal size is small (<1 μm, in radius), the macrophage lysosomes can undergo biogenesis^47,48^. A master regulator of lysosomal biogenesis and V-ATPase expression is transcription factor EB (TFEB)^39,46,49^. Moreover, given the recent findings regarding the role of TFEB in upregulating lysosomal genes during inflammation^50^, the role of TFEB in the bioaccumulation and stabilization of CFZ-H^+^Cl^−^ salt in macrophage lysosomes, in relation to CFZ’s anti-inflammatory activity^9,10^, could be further investigated.

To conclude, we have discovered how lysosomal ion transport pathways play a major role in the self-assembly of a biocompatible, cell-targeted supramolecular structure formed by a weakly basic, small molecule building block. Together with the physicochemical properties of the freely soluble building blocks, these transport pathways can be exploited to develop novel types of drug depots and cell-directed drug delivery systems^51–53^. Indeed, interest has been raised in macrophages as drug delivery vehicles. As our studies have shown, macrophages may allow drugs to be locally stabilized and undergo a sustained release in a site-specific manner, offering a strategy for reducing off-target side effects^54,55^. Macrophages are also one of the few cell types, which are primarily involved in the phagocytosis of foreign particles of different sizes and shapes, including drugs^56,57^, which offer yet another opportunity for loading these cells with massive amounts of chemotherapeutic agents. To this end, additional investigations into the manner in which these cells mechanically synergize with the self-assembled weakly basic small molecule building blocks in order to stabilize functional mechanopharmaceutical devices are warranted.

## Electronic supplementary material

Supplementary Information
