# Supplementary material for: Reverse Engineering the Intracellular Self-Assembly of a Functional Mechanopharmaceutical Device
Source: Sci Rep. 2018 Feb 13;8:2934. doi: 10.1038/s41598-018-21271-7 (PMC5811454; doi:10.1038/s41598-018-21271-7)
Supplement: Supplementary file 1 — Supplementary Information [file 41598_2018_21271_MOESM1_ESM.pdf]

Reverse Engineering the Intracellular Self-Assembly of a Functional Mechanopharmaceutical Device

Tehetina Woldemichael[1#], Rahul K. Keswani[2#], Phillip Rzeczycki[2], Mikhail D. Murashov[2], Vernon LaLone[2], Brian Gregorka[3], Joel A. Swanson[4], Kathleen A. Stringer[5], and Gus R. Rosania[2*]

[1]Biophysics Program, College of Literature, Science, and the Arts, University of Michigan, Ann Arbor, MI, USA

[2]Department of Pharmaceutical Sciences, College of Pharmacy, University of Michigan, Ann Arbor, MI, USA

[3]CLCI: Center for Live-Cell Imaging, Department of Microbiology and Immunology, University of Michigan, Ann Arbor, MI, USA

[4]Program in Immunology and Department of Microbiology and Immunology, University of Michigan Medical School, Ann Arbor, MI, USA

[5]Department of Clinical Pharmacy, College of Pharmacy, University of Michigan, Ann Arbor, MI, USA

* Corresponding author
Email: grosania@umich.edu

#These authors contributed equally to this work

Supplementary Methods

HPLC analysis. For the HPLC assay, the mobile phase was methanol:water (80:20) with 0.1% trifluoroacetic acid (1 ml/min flow rate). The stationary phase (column) was a C18 (unbonded silica particles) column (Atlantis® T3, 5 μm, 100Å), and the HPLC was equipped with a UV detector (Waters, Photodiode Array Detector 2996) @ 285 nm detection for CFZ. The retention time for CFZ was determined to be 4.75 min.

Determination of solubility parameters. We used a mathematical proof approach to determine $[CFZH^{+}]_S$ as detailed below:

First, we calculated $[CFZH^{+}]$ using the total solubility (S_T) at each pH value and the constant intrinsic free base solubility, $[CFZ]_S$, using the following equation, which was obtained by rearranging the terms in equation (14) (see main article):

$$[\mathrm{CFZH}^{+}] = S_{\mathrm{T}} - [\mathrm{CFZ}]_{\mathrm{S}} \qquad \text{(Supplementary Equation 1)}$$

Then, we chose a value, say "y", from the list of the computed $[CFZH^{+}]$ values, and let "y" equal $[CFZH^{+}]_S$. By substituting "y' in place of $[CFZH^{+}]_S$, we calculated for total solubility at the different pH values using equation (15) (see main article). Then, we compared this total solubility-pH dataset to that obtained using equation (13) (see main article). Then, we checked if there was the same total solubility value at a given pH in both datasets, which would represent the intersection point of the two solubility-pH curves mentioned previously. This can be represented by the following equation:

$$S_{\mathrm{T}}{}' = [\mathrm{CFZ}]_{\mathrm{S}} + [\mathrm{CFZH}^{+}]_{\mathrm{S}} \qquad \text{(Supplementary Equation 2)}$$

Where S_T' is the total solubility value at the intersection of the two solubility-pH curves, and both forms of the drug, CFZ and $CFZH^+$, are present in equilibrium with their respective solid forms in the solid phase denoted by the subscript *s*.

Moreover, to prove if our earlier assumption ("y" is equal to $[CFZH^+]_S$) was valid, we calculated the total solubility by plugging "y" in place of $[CFZH^+]_S$, supplementary equation (2), and checked if it was equal to S_T'. Furthermore, by definition, the pH where both the intrinsic free base and salt forms of the drug are in the solid phase is known as pH_{max}. Thus, the pH associated with S_T' was deduced to be pH_{max}.

Modeling proton influx. V-ATPase is an electrogenic proton-pump, which inserts protons from the cytoplasm into the lysosome against an electrochemical gradient upon ATP hydrolysis[1,2]. The rate at which a proton molecule is inserted per second (J_{HVATP}) was obtained from published experimental studies[1] as a function of transmembrane pH gradient (Δ pH) in units of pH unit and membrane potential difference, which is also interchangeably known as membrane potential (ΔΨ), in units of mV. This rate was multiplied by the total number of active V-ATPase molecules per lysosome (N_{VATP}) to obtain the total amount of proton molecules inserted into the lysosome in units of molecules per second, as follows:

$$H_{pump} = N_{VATP} \times J_{H_{VATP}} \, (\Delta \, pH \, , \, \Delta \, \Psi) \qquad \text{(Supplementary Equation 3)}$$

Where lysosomal ΔΨ is dictated by the total net change in lysosomal ion content and is represented by the following relationship[3]:

$$\Delta \, \Psi = \frac{F \times V_L}{C' \times S} \times [\, (\textstyle\sum_i Z_i [cations]_i + \sum_i Z_i [anions]_i \,) - B] \qquad \text{(Supplementary Equation 4)}$$

Where V_L is the lysosomal volume in units of L; Faraday's constant (F) which equals 96485 Coulomb/mol and is used to convert the lysosomal ion content in units of moles to units of Coulomb; C' is the specific membrane capacitance per unit area of a biological membrane[4], which is experimentally approximated to be $1\mu F/cm^2$ and is multiplied by the lysosomal surface area S represented in units of cm^2 to obtain the total lysosomal membrane capacitance; Z_i is valence for ion i; [cation] the concentration of cation i at a given time *t* in units of Molar; [anion] is the concentration of anion i at a given time *t* in units of Molar; B is the Donnan particles in units of Molar, which are impermeable lysosomal contents defined by initial lysosomal ion concentrations and net change in the intrinsic surface potentials, supplementary equation (5). In the model and simulations communicated herein, the initial lysosomal ions consist of proton, potassium, sodium, and chloride with their respective charge (+ or -1) represented as a coefficient of their respective concentration, which is denoted by the square brackets, supplementary equation (5).

$$B = [H^+]_{L,initial} + [K^+]_{L,initial} + [Na^+]_{L,initial} - [Cl^-]_{L,initial} - \frac{C\times S}{F\times V_L} \times \{(\Psi_{in} - \Psi_{out}) + \Psi_{initial}\} \quad \text{(Supplementary Equation 5)}$$

Where Ψ_{in} and Ψ_{out} are the intrinsic inner and outer surface potentials, respectively, in units of mV, which contribute to the change in ion concentration at membrane surface as described later, $\Psi_{initial}$ is the initial lysosomal membrane potential which is set to zero mV in order to maintain initial lysosomal membrane electroneutrality.

Modeling chloride influx. V-ATPase mediated proton influx into the lysosome is followed by lysosomal membrane potential increase which consequentially arrests further proton influx. Thus, to lower the membrane potential for the continuation of V-ATPase proton-pumping

activity, which is essential to lower lysosomal pH to physiological pH, the removal of lysosomal cation or the insertion of lysosomal anion is required. Accordingly, CLC7 is considered as the primary membrane potential dissipating protein which transports two chloride ions from the cytoplasm to the lysosome for every proton it transports from the lysosome to the cytoplasm[5]. The rate ($J_{Cl,\ HClC7}$) at which the ion transportations occur was empirically derived from a current-voltage experimental data[3] as a function of chemical (ΔpH, ΔCl) and electric potential gradients, ($\Delta\Psi$), as detailed in supplementary equations (6) and (7). This rate was multiplied by the total number of CLC7 molecules per lysosome (N_{ClC7}) in order to obtain the total amount of proton and chloride molecules transported across the lysosomal membrane through CLC7, as follows:

$$H_{CLC7} = N_{CLC7} \times J_{Cl,H_{CLC7}}(\Delta\,pH,\ \Delta\,Cl,\ \Delta\,\Psi) \qquad \text{(Supplementary Equation 6)}$$

$$Cl_{CLC7} = 2 \times N_{CLC7} \times J_{Cl,H_{CLC7}}(\Delta\,pH,\ \Delta\,Cl,\ \Delta\,\Psi) \qquad \text{(Supplementary Equation 7)}$$

Where ΔCl is the chloride gradient comprised of the luminal chloride (Cl_L) and the cytoplasmic chloride (Cl_C). Moreover, the coefficient 2 in supplementary equation (7) defines the 2:1 stoichiometric relationship between the chloride and proton ions transported by CLC7 across the lysosomal membrane.

Modeling proton efflux. In addition to CLC7, the passive diffusion of protons across the lysosomal membrane can also contribute to the dissipation of membrane potential in order to facilitate the proton pumping activity of V-ATPase. This passive proton leak is modeled by supplementary equation (8), which is derived from the Goldman-Hodgkin-Katz (GHK) ion flux equation[6] that is commonly used to describe the passive diffusion of a given ion across a biological membrane, assuming a linear potential gradient across a lipid membrane[3].

$$H_{leak} = \left(S \times P_{H^+} \times Y \times Z \times \frac{10^{-pH_L} - \left(10^{-pH_C} \times e^{-Z\times Y}\right)}{1 - e^{-Z\times Y}}\right) \times N_{av} \qquad \text{(Supplementary Equation 8)}$$

Where S is the total lysosomal surface area in units of cm^2 used to obtain the total amount of proton which passively diffuses across the lysosomal membrane; P_H^+ is the lysosomal membrane proton permeability in units of cm/s; Z is the valence of the ion (i.e. +1 for proton); pH_L is the luminal pH used to calculate the total free lysosomal proton based on the logarithmic relationship of pH and free proton ($pH_L = -\log[H^+]$); pH_c is the cytoplasmic pH used to calculate the total free cytoplasmic proton based on the logarithmic relationship of pH and free proton ($pH_C = -\log[H^+]$); N_{av} is Avogadro's number used to convert the amount of transported protons in unit of moles to unit of molecules; Y is used to convert proton transportation in unit of charge per second to moles per second and is defined as: $Y = \frac{\Delta\,\Psi \times F}{R \times T}$, where R is universal gas constant, F is Faraday's constant, and T is absolute temperature. Moreover, for cells at room temperature (25 °C), RT/F equals 25.69 mV[4], and is used for normalizing the lysosomal membrane potential communicated in this report.

Modeling the effect of membrane leaflet potentials on ions. In order to account for the effects of intrinsic external ($\Psi_{i,out}$) and internal ($\Psi_{i,in}$) leaflet potentials of the lysosomal membrane on cytoplasmic and lysosomal ion *i* concentrations, respectively, the individual cytoplasmic and lysosomal ion concentrations are computed using the following relationships, supplementary equations (9 and 10), derived from the GHK equation for a single ion concentration gradient, by setting net current flow equal to zero.

$$\Delta\,\Psi_{i,in} = \frac{-Z \times R \times T}{F} \ln \frac{C_{i,in}}{C_{i,L}} \qquad \text{(Supplementary Equation 9)}$$

$$\Delta\,\Psi_{i,out} = \frac{-Z \times R \times T}{F} \ln \frac{C_{i,out}}{C_{i,C}} \qquad \text{(Supplementary Equation 10)}$$

Where $C_{i,in}$ is the internal concentration of a given ion (i) at the membrane surface facing the lysosomal compartment, $C_{i,L}$ is the concentration of the ion (i) inside the lysosome, $C_{i,out}$ is the external concentration of the ion (i) at the membrane surface facing the cytoplasmic compartment, $C_{i,C}$ is the concentration of the ion (i) inside the cytoplasm. All units are molar.

Governing equations. Supplementary equations (3-10) were used in supplementary equations (11-13) to further define the ion movements as a function of time, in units of molecules per second.

$$\frac{dH^+}{dt} = H_{pump} - H_{CLC7} - H_{leak} - H_{sequestered} \qquad \text{(Supplementary Equation 11)}$$

$$\frac{dCl^-}{dt} = 2 \times N_{CLC7} \times J_{Cl,H_{CLC7}}(\Delta pH, \Delta Cl, \Delta \Psi) - Cl_{sequestered}$$

(Supplementary Equation 12)

Moreover, extending supplementary equation (11), we can denote the change in lysosomal pH with respect to time by following the relationship between the lysosomal lumen buffering capacity (β) of the Donnan particles, which sequester non-free lysosomal protons, and free lysosomal protons which give rise to lysosomal pH as follows:

$$\frac{dpH}{dt} = \frac{(-H_{pump} + H_{CLC7} + H_{leak} + H_{sequestered})}{V \times N_{av} \times \beta} \qquad \text{(Supplementary Equation 13)}$$

Where V is the lysosomal volume and is multiplied by N_{av} to convert the unit of molecules per second to molar per second, where the inverse of β is in units of pH units per molar.

Numerical analysis. Supplementary equations (11-13) were solved by numerical integration in Berkeley Madonna® using Rosenbrock stiff solver as a numerical integrator.

Data visualization. Multiple individual 2D datasets associated with the parametric simulations performed to generate simultaneous inhibitions of various lysosomal parameters were obtained. The datasets associated with each simultaneous inhibitions of lysosomal parameters were exported from Berkeley Madonna and compiled into three separate matrices in a MS-Excel spreadsheet, such that the first rows and columns of the matrix correspond to the two parameters simultaneously varied in the model simulations to obtain the final lysosomal readout values (lysosomal pH, Cl^-, and membrane potential), where each makes up the rest of the rows and columns of a single matrix.

References

1 Grabe, M., Wang, H. & Oster, G. The mechanochemistry of V-ATPase proton pumps. *Biophys J* **78**, 2798-2813, doi:10.1016/S0006-3495(00)76823-8 (2000).

2 Grabe, M. & Oster, G. Regulation of organelle acidity. *J Gen Physiol* **117**, 329-344 (2001).

3 Ishida, Y., Nayak, S., Mindell, J. A. & Grabe, M. A model of lysosomal pH regulation. *J Gen Physiol* **141**, 705-720, doi:10.1085/jgp.201210930 (2013).

4 Hille, B. *Ionic Channels of Excitable Membranes. 2nd Edition*. 607 (Sinauer Associates, Inc. , 1992).

5 Graves, A. R., Curran, P. K., Smith, C. L. & Mindell, J. A. The Cl-/H+ antiporter ClC-7 is the primary chloride permeation pathway in lysosomes. *Nature* **453**, 788-792, doi:10.1038/nature06907 (2008).

6 Weiss, T. F. *Cellular Biophysics: Transport*. (MIT Press, 1996).

7 Van Dyke, R. W. Acidification of rat liver lysosomes: quantitation and comparison with endosomes. *Am J Physiol* **265**, C901-917 (1993).

8 Gambale, F., Kolb, H. A., Cantu, A. M. & Hedrich, R. The Voltage-Dependent H+-Atpase of the Sugar-Beet Vacuole Is Reversible. *Eur Biophys J Biophy* **22**, 399-403 (1994).

9 Heuser, J., Zhu, Q. & Clarke, M. Proton pumps populate the contractile vacuoles of Dictyostelium amoebae. *J Cell Biol* **121**, 1311-1327 (1993).

10 Sonawane, N. D., Thiagarajah, J. R. & Verkman, A. S. Chloride concentration in endosomes measured using a ratioable fluorescent Cl- indicator - Evidence for chloride accumulation during acidification. *Journal of Biological Chemistry* **277**, 5506-5513, doi:10.1074/jbc.M110818200 (2002).

11 Alberts, B. J., A.; Lewis, J.; Raff, M.; Roberts, K.; Walter, P. *Moelcular Biology of the Cell. Fifth edition*. (Garland Science, New York, 2008).

Supplementary Figures and Legends

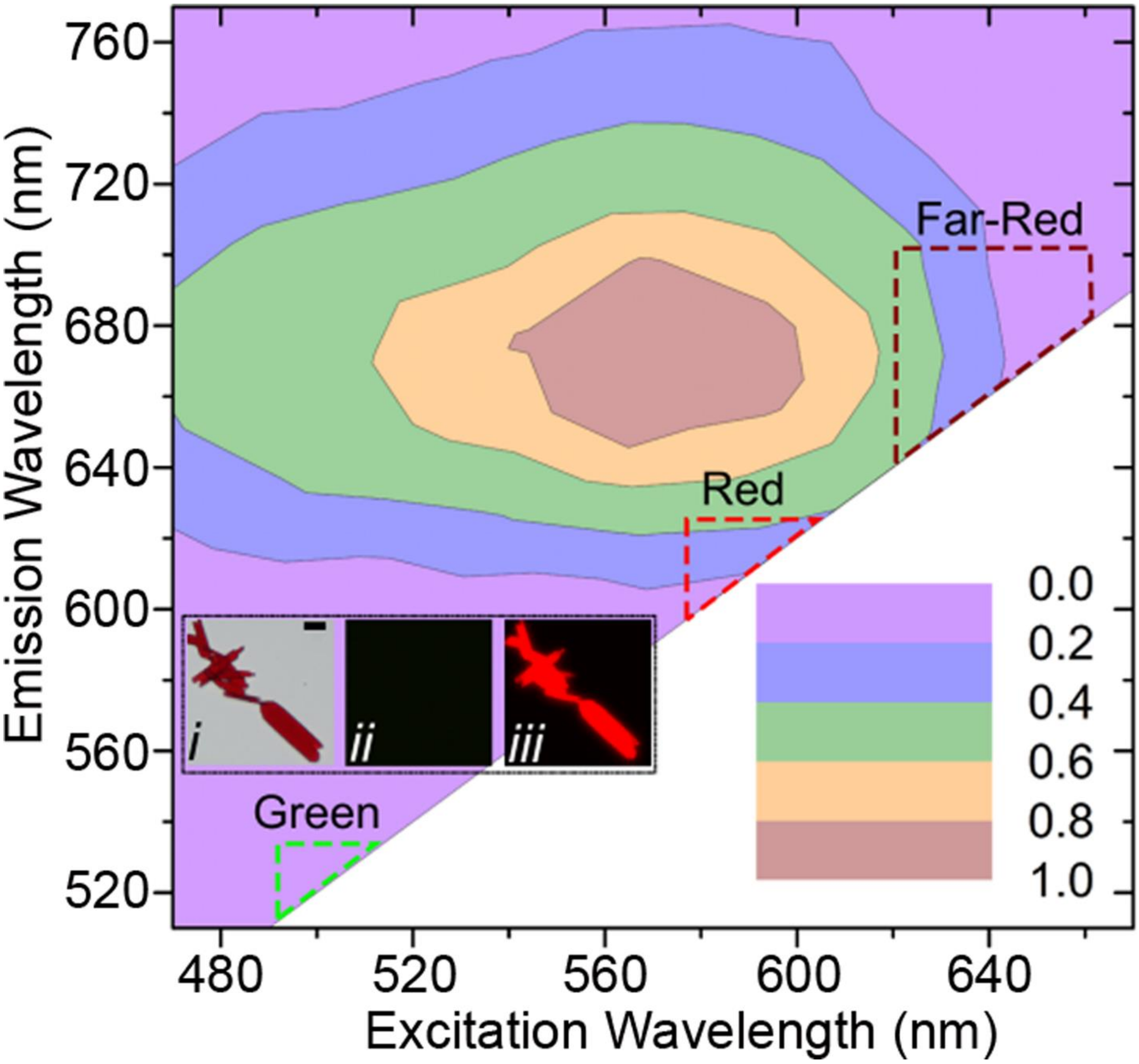

Supplementary Figure S1. Fluorescence Microspectroscopy and (inset, *i* = brightfield, *ii* = green fluorescence, *iii* = far-red fluorescence) Epifluorescence Microscopy of CFZ-H^+Cl^- crystals indicating the lack of green fluorescence while being fluorescent in the far-red fluorescence range. The excitation wavelength (nm, Ex) and emission wavelength (nm, Em) are shown on the X-axis and Y-axis, respectively. The normalized fluorescence yield is shown by a

contour plot that was normalized using a control slide first and then to the maximum measured fluorescence yield. The colors as shown represent contour levels from 0-1 in steps of 0.2. The green, red, and far-red fluorescence bandwidths are overlaid on top of the normalized fluorescence spectra.

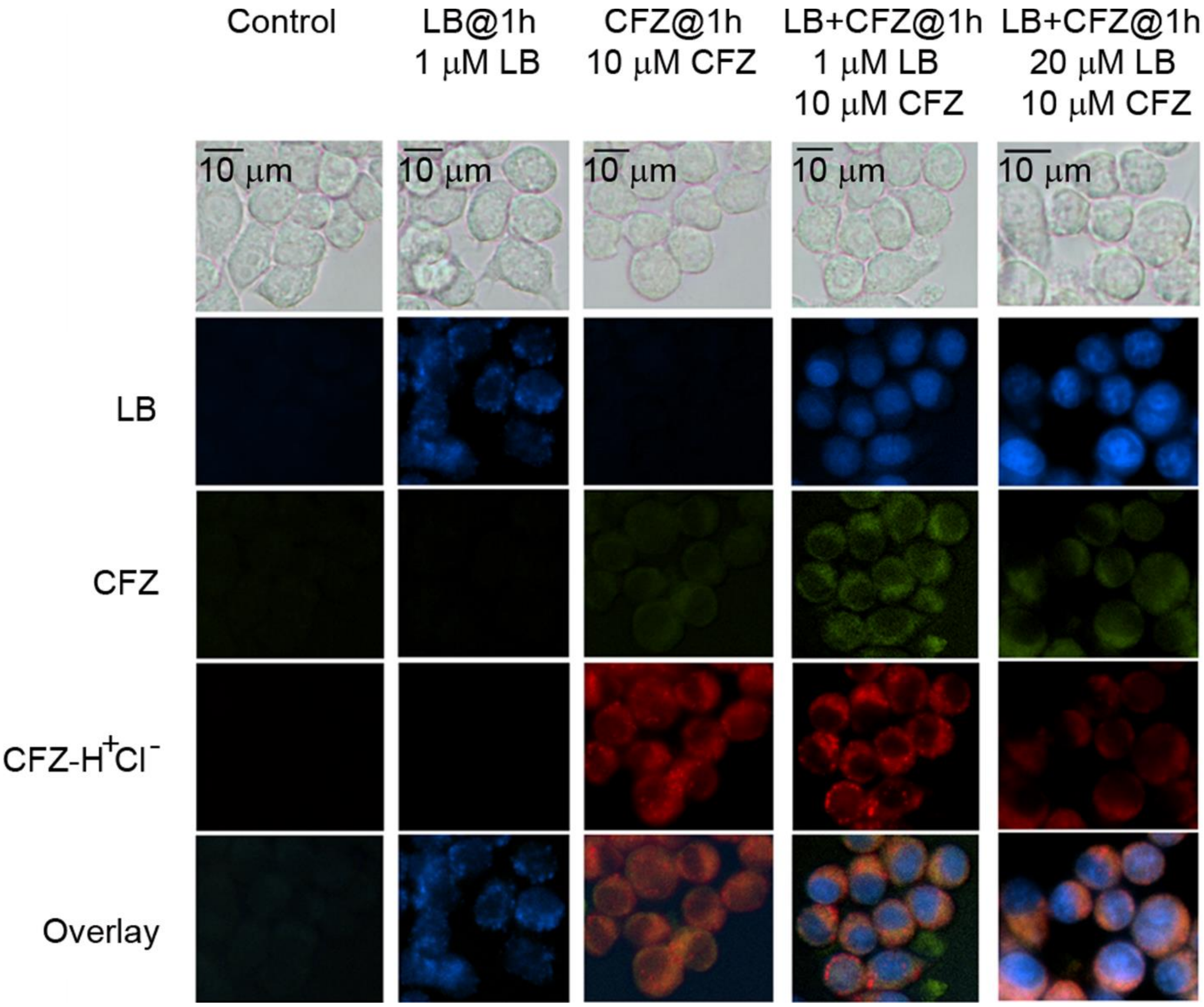

Supplementary Figure S2. Epifluorescence microscopy of RAW264.7 cells when incubated with CFZ (10 μM), Lysotracker® Blue (LB, 1 and 20 μM) at t = 1 hour. Scale bar, 10 μm. At 1 hour, green fluorescence spots indicative of CFZ are visible as mild diffuse staining rather than punctate staining observed at t = 24 hours. Vesicular staining pattern of LB, visible as blue

punctate spots when incubated on its own, are absent when co-incubated with CFZ at both LB = 1 and 20 μM. The control images were taken at t = 24 hours post initiation of experiments.

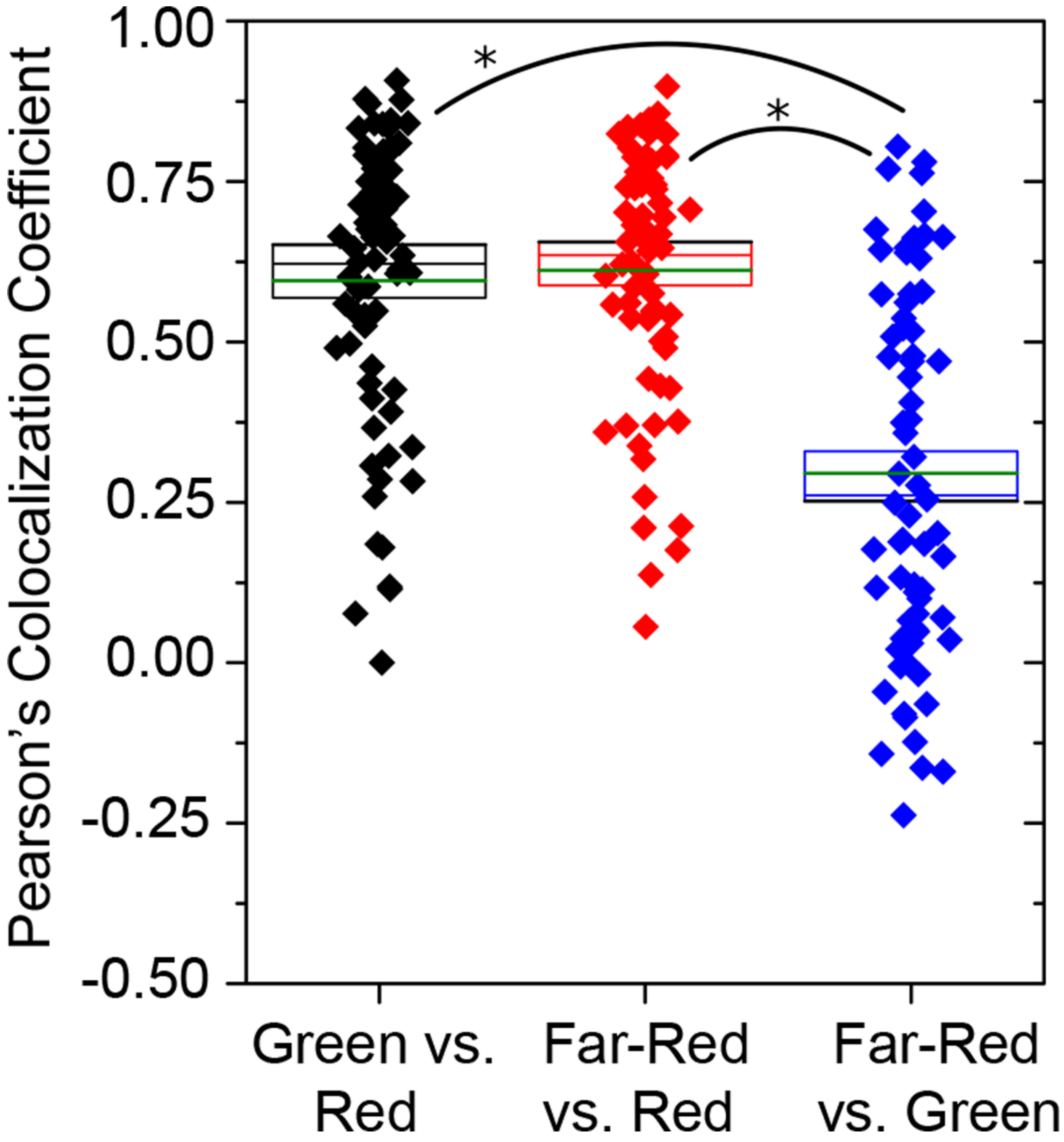

Supplementary Figure S3. Pearson's Colocalization Coefficient for multiple cell ROIs (n = 70) as obtained post epifluorescence microscopy of RAW264.7 cells incubated with CFZ (10 μM) at t = 24-72 hours. Fluorescence channel description is provided in Supplementary Table S2. The scatter distribution is further annotated with four horizontal lines denoting the Mean ± S.E. (same color lines as the distribution), Mean (green line) and the Median (black line) of the distribution. * -$p < 0.005$.

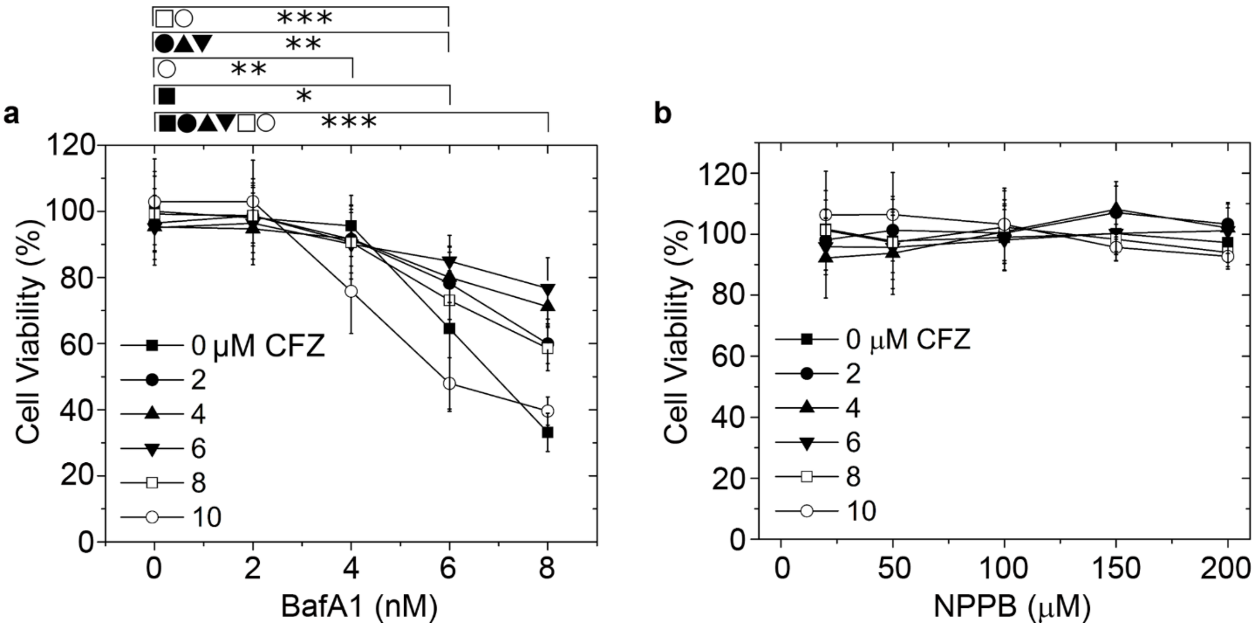

Supplementary Figure S4. Viability of RAW264.7 cells treated with a) BafA1 and b) NPPB in the presence of CFZ (0-10 μM). Data was collected at n = 6 with * - $p < 0.05$, ** - $p < 0.01$, *** - $p < 0.001$. Each point is compared in a pair-wise independent Student t-test.

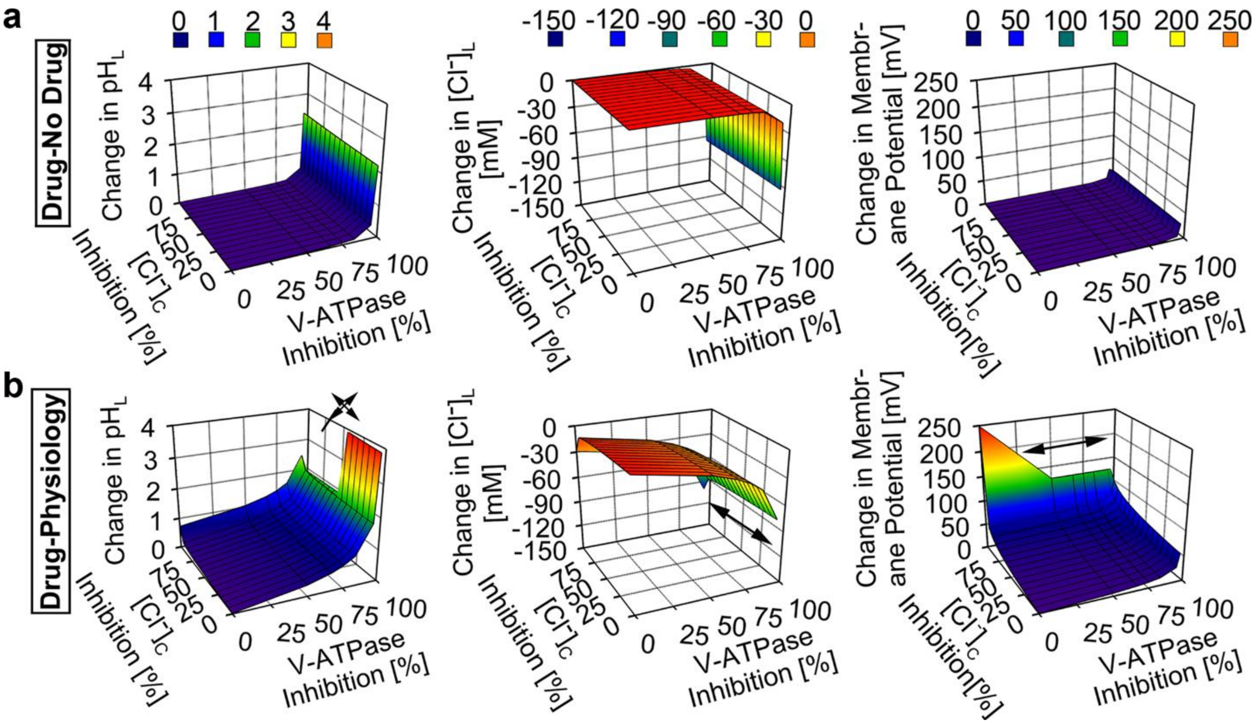

Supplementary Figure S5. **Model and simulation of the effects of V-ATPase and cytoplasmic chloride on the lysosomal accumulation of CFZ-H$^+$Cl$^-$. a**, V-ATPase inhibition showed a more substantial effect than cytoplasmic chloride inhibition on the accumulation of CFZ-H$^+$Cl$^-$ at the rate of 0.01 picomol/cell/day, as reflected by the changes in the lysosomal pH, Cl$^-$, and membrane potential values of the CFZ-H$^+$Cl$^-$ containing lysosome from that of the CFZ-H$^+$Cl$^-$ free lysosome. **b**, V-ATPase inhibition generally showed a more substantial effect than cytoplasmic chloride inhibition although the simultaneous inhibition of both parameters showed even more pronounced effect on the physiological accumulation of CFZ-H$^+$Cl$^-$ at the rate of 0.01 picomol/cell/day, as reflected by the changes in the lysosomal pH, Cl$^-$, and membrane potential values of the CFZ-H$^+$Cl$^-$ containing lysosome from respective baseline physiological values. Arrow signs represent values outside of the axes plot range.

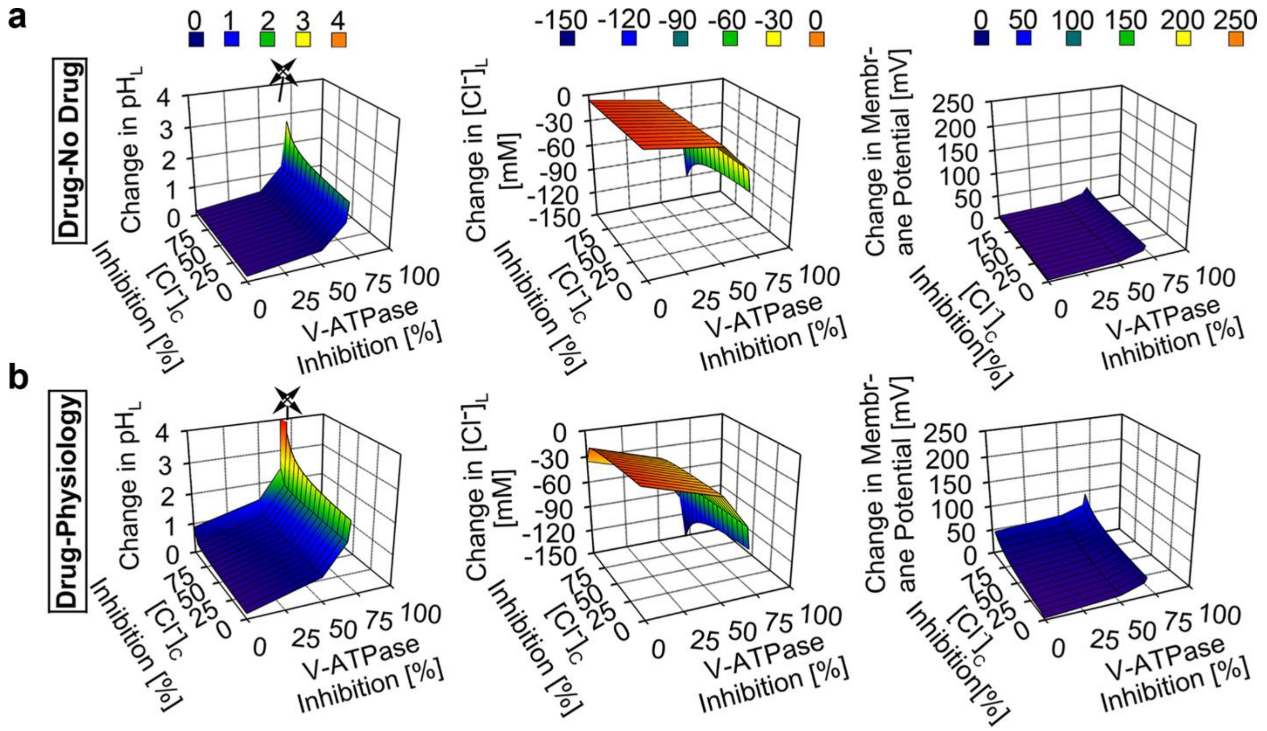

Supplementary Figure S6. Model and simulation of the effects of V-ATPase and cytoplasmic chloride on the lysosomal accumulation of CFZ-H^+Cl^- at a higher dose. a, V-ATPase inhibition showed a more substantial effect than cytoplasmic chloride inhibition on the accumulation of CFZ-H^+Cl^- at the rate of 0.1 picomol/cell/day, as reflected by the changes in the lysosomal pH, Cl^-, and membrane potential values of the CFZ-H^+Cl^- containing lysosome from that of the CFZ-H^+Cl^- free lysosome. **b**, V-ATPase inhibition generally showed a more substantial effect than cytoplasmic chloride inhibition although the simultaneous inhibition of both parameters showed even more pronounced effect on the physiological accumulation of CFZ-H^+Cl^- at the rate of 0.1 picomol/cell/day, as reflected by the changes in the lysosomal pH, Cl^-, and membrane potential values of the CFZ-H^+Cl^- containing lysosome from respective baseline physiological values. Arrow signs represent values outside of the axes plot range.

Supplementary Table S1. Model Parameters

Symbol	Description	Baseline Input Value	Range of Input Value	Units
pH_C	Cytosolic pH	7.2	Fixed	pH Unit
pH_L	Luminal pH	7.4	Fixed	pH Unit
$[Cl^-]_C$	Cytosolic chloride concentration	10	$1x10^{-5}$ - 10	mM
$[Cl^-]_L$	Luminal chloride concentration	110	Fixed	mM
$[Na^+]_L$	Luminal sodium concentration	145	Fixed	mM
$[K^+]_L$	Luminal potassium concentration	5	Fixed	mM
$[H^+]_L$	Luminal proton concentration	0	Fixed	mM
P_{H^+}	Membrane proton permeability	$6x10^{-5}$	Fixed	cm/s
V	Lysosomal volume	$1.65x10^{-16}$	Fixed	L
S	Lysosomal surface area	$1.45x10^{-8}$	Fixed	cm^2
C'	Specific bilayer capacitance	1	Fixed	$\mu Farad/cm^2$
β	Buffering capacity	40	Fixed	mM/pH unit
N_{VATP}	V-ATPase number	300	$1x10^{-4}$ - 300	
N_{CLC7}	CLC7 number	5000	$1x10^{-4}$ - 5000	
Ψ_{out}	Outer surface potential	-50[b]	Fixed	mV
Ψ_{in}	Inner surface potential	0[b]	Fixed	mV
CLC_Cl	CLC7 Cl^- stoichiometry	2	Fixed	
CLC_H	CLC7 H^+ stoichiometry	1	Fixed	
R	Gas constant	8.314	Fixed	$J.K^{-1}.mol^{-1}$
T	Absolute temperature	0	Fixed	Kelvin
F	Faraday's constant	96485	Fixed	J/Volt
N_{av}	Avogardro's number	$6.02x10^{23}$	Fixed	molecules/mol
a	Rate of proton and chloride sequestration by CFZ to form $CFZ\text{-}H^+Cl^-$	0	$1.16x10^{-21}$ - $1.16x10^{-20}$	Moles/day

[a] Baseline input values are literature values[7-11] representing physiological lysosomes and are in agreement with previously published model[2,3].

[b] Estimated intrinsic surface potentials for inner (Ψ_{in}) and outer (Ψ_{out}) leaflets of the lysosomal membrane accounted for when modeling membrane transporter mediated dynamic lysosomal and cytoplasmic ion concentrations at the membrane surface[2].

Supplementary Table S2. Fluorescence in RAW264.7 cells. (Channels (Ex/Em)) – Blue (350/405 nm), Green (490/510 nm), Red (590/610 nm), and Far-Red (640/670 nm).

	**Blue**	**Green**	**Red**	**Far-Red**
**CFZ**	-	+	+	-
**CFZ-H^+Cl^-**	-	-	+	+
**Lysotracker® Blue**	+	-	-	-
